# RNA-based translation activators for targeted gene upregulation

**DOI:** 10.1038/s41467-023-42252-z

**Published:** 2023-10-26

**Authors:** Yang Cao, Huachun Liu, Shannon S. Lu, Krysten A. Jones, Anitha P. Govind, Okunola Jeyifous, Christine Q. Simmons, Negar Tabatabaei, William N. Green, Jimmy. L. Holder, Soroush Tahmasebi, Alfred L. George, Bryan C. Dickinson

**Affiliations:** 1 Department of Chemistry, The University of Chicago, Chicago, IL USA; 2 Department of Neurobiology, The University of Chicago, Chicago, IL USA; 3 Department of Pharmacology, Northwestern University Feinberg School of Medicine, Chicago, IL USA; 4 Department of Pharmacology and Regenerative Medicine, University of Illinois College of Medicine, Chicago, IL USA; 5 Department of Pediatrics, Baylor College of Medicine, Houston, TX USA; 6 Jan and Dan Duncan Neurological Research Institute, Texas Children’s Hospital, Houston, TX USA

**Keywords:** Synthetic biology, RNA, Translation, Ribosome, Gene regulation

## Abstract

Technologies capable of programmable translation activation offer strategies to develop therapeutics for diseases caused by insufficient gene expression. Here, we present “translation-activating RNAs” (taRNAs), a bifunctional RNA-based molecular technology that binds to a specific mRNA of interest and directly upregulates its translation. taRNAs are constructed from a variety of viral or mammalian RNA internal ribosome entry sites (IRESs) and upregulate translation for a suite of target mRNAs. We minimize the taRNA scaffold to 94 nucleotides, identify two translation initiation factor proteins responsible for taRNA activity, and validate the technology by amplifying SYNGAP1 expression, a haploinsufficiency disease target, in patient-derived cells. Finally, taRNAs are suitable for delivery as RNA molecules by lipid nanoparticles (LNPs) to cell lines, primary neurons, and mouse liver in vivo. taRNAs provide a general and compact nucleic acid-based technology to upregulate protein production from endogenous mRNAs, and may open up possibilities for therapeutic RNA research.

## Abstract (web summary)

Many diseases are driven by the insufficient expression of critical genes, but few technologies are capable of rescuing these endogenous protein levels. Here, Cao et al. present an RNA-based technology that boosts protein production from endogenous mRNAs by upregulating their translation.

## Introduction

Insufficient expression of critical proteins, triggered by gene deletions, mutations, or expression downregulation by pathological conditions, drive a large swath of human diseases, including cancer^1^, neurodegeneration^2^, metabolic disorders^3^, and rare genetic disease^4,5^. Despite the therapeutic need, technologies to *activate* gene expression have been underdeveloped. Recent innovations in chemical modifications^6^ and delivery methods^7^ of nucleic acid-based therapeutics (NBTs) have driven clinical successes^8^, and opened new possibilities for gene-activation technologies^9^. Broadly, these involve mRNA delivery^10^, splicing modulators^8,11^, transcript stabilizers^12^ or activators^13,14^, and translation activators^15–18^. Technologies that increase productive RNA levels, including TANGO (targeted augmentation of nuclear gene output), which prevents the inclusion of toxic exons^19^; AntagoNATs (single-stranded NAT-specific oligonucleotides), which inhibit natural antisense transcripts^20^; and saRNAs (small activating RNAs), which are double-stranded RNAs to activate DNA transcription^21^, are either advancing towards or have already reached clinical stages^9^. Compared to the overall successes of transcript-regulating technologies, those that directly modulate protein translation are comparably lagging.

Translation activators, which upregulate protein production from cellular mRNAs, do not rely on specific transcriptional regulatory properties, therefore should be suitable for a broad range of mRNAs. Existing antisense oligonucleotides (ASOs)-based translation activators, which block either upstream AUG regions^15^ or inhibitory elements in 5′ UTRs^16^, have potential, but cannot target transcripts without such specific regulatory elements. Long-noncoding RNA (lncRNA)-based translation activators are generally 250~1200 nt^18,22^, making them challenging to manufacture as chemically modified systems, which limits delivery optons. Importantly, lncRNA-based translation activators require binding domains that overlap the translation initiation sites on target transcripts^22^, and are thus unsuitable for transcripts with no suitable binding sites near start codon, due to either having short 5′ UTRs or critical off-targets based on that limited choice of sites.

**Fig. 1:**
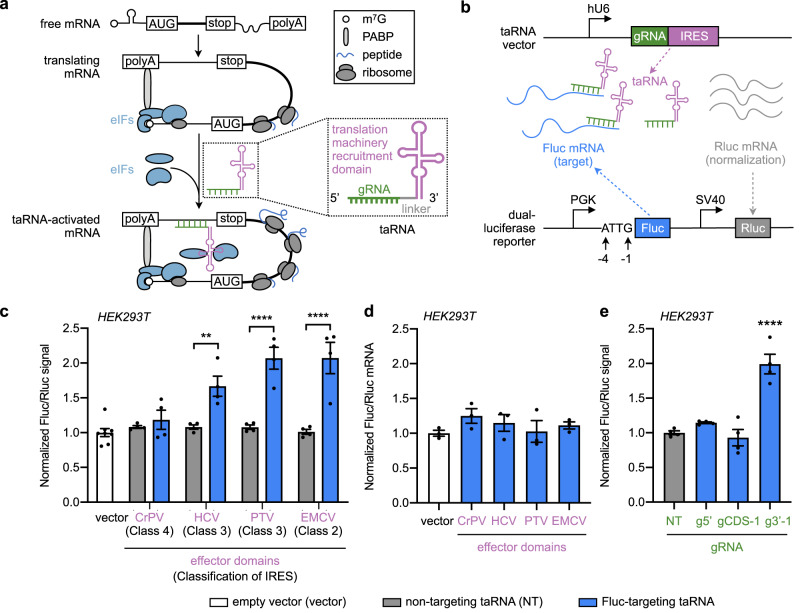
taRNAs built from an array of IRESs increase reporter gene translation. **a** Schematic overview of taRNA technology. taRNAs recruit initiation factors (eIFs, blue) to increase targeted mRNA translation. A taRNA molecule is made of a target-specific guide RNA domain (gRNA, green), a linker (gray) and a translation machinery-recruitment domain (purple). Poly(A)-binding protein (PABP, light gray) interacts with both eIFs and mRNA poly-A tail. **b** Schematic of vectors for taRNA and reporter used in dual-luciferase assay. The taRNA (green and purple) targets Firefly luciferase mRNA (Fluc, blue), while Renilla luciferase (Rluc, gray) is the internal control. The nucleotides at position −4 to −1 before the start codon of Fluc mRNA are ATTG. The hU6 (human U6), PGK (phosphoglycerate kinase 1) and SV40 (simian vacuolating virus 40) indicate different promoters. **c** Evaluation of viral IRESs as taRNA effector domains in HEK293T cells by dual-luciferase assay. Each IRES was attached to either a non-targeting gRNA (gray), or an Fluc-targeting gRNA (g3′-1, blue) on the taRNA vector, and co-transfected into HEK293T cells with the reporter. Viral origin and classification are listed for each IRES. Data were normalized to empty vector group. *n* = 8 biological replicates for empty vector group. *n* = 4 biological replicates for all the other groups. **d** RNA level of Fluc relative to Rluc in HEK293T cells after taRNA treatment (gRNA: g3′ -1, blue) measured by RT-qPCR. Data were normalized to empty vector group. *n* = 3 biological replicates. **e** PTV-based taRNAs with gRNAs that bind to different regions on Fluc transcript, including 5′ UTR (g5′), CDS (gCDS-1) and 3′ UTR (g3′-1), were evaluated by dual-luciferase assay. *n* = 4 biological replicates. All data are shown as mean ± SEM with individual data points. **c** Statistical analyses were performed using two-way ANOVA with Sidak’s multiple comparisons test between non-targeting and Fluc-targeting within each IRES group. Statistical analyses were performed using one-way ANOVA with Dunnett’s multiple comparison test **d** vs. vector; **e** vs. NT. **P < 0.01, ****P < 0.0001. No asterisk = not significant. The *P* value and source data are provided as a Source Data file.

Here, we describe the development of “translation-activating RNAs” (taRNAs), a programmable RNA-based platform that directly upregulates translation of wide-ranging mRNAs of interest in mammalian systems both in vitro and in vivo. taRNAs are bifunctional molecules, made from a guide sequence that can be flexibly chosen from 3’ UTR-binding sites on a target mRNA, and an effector domain that recruits translation machinery (Fig. 1a). The effector domain can be selected from a collection of RNA elements, which were mined from natural internal ribosome entry sites (IRESs)^23–25^. We demonstrate that taRNAs can increase protein synthesis from an array of mRNAs of interest in human and mouse cells, including haploinsufficiency-disease-related targets such as SYNGAP1 and PMP22. Our engineering efforts revealed eIF3 and eIF4G as the key initiation factors recruited to boost targeted mRNA translation, and minimized the taRNA scaffold to 94 nucleotides. Naked taRNAs packaged in lipid nanoparticles (LNPs) can be delivered to primary rat cortex neurons in vitro and to mouse liver in vivo. Importantly, we validated the therapeutic potential of taRNAs by restoring SYNGAP1 expression in iPSC-neurons generated from an individual with *SYNGAP1* haploinsufficiency. Collectively, the taRNA technology provides a customizable RNA-based platform for elevating gene expression from various endogenous mRNAs, opening up possibilities for therapeutic design.

## Results

### Design principles of taRNA platform

To identify suitable RNA elements as taRNAs’ effector domains, we mined a collection of IRESs, which can promote eukaryotic mRNA translation initiation by directly recruiting eukaryotic translation initiation factors (eIFs)^26^. Initiation is typically the rate-limiting step of mRNA translation^27^, and recruiting eIFs to the 5’ or 3’ UTR naturally^28^, or artificially by RNA-targeting technologies^29–31^, has been shown to enhance target protein production. We hypothesized that IRESs could be encoded separately as taRNAs to recruit eIFs in *trans* when delivered to an mRNA of interest by a guiding sequence (Fig. 1a), although IRESs are only found and utilized as *cis*-acting elements to regulate translation in known natural systems^25,32^.

To test initial taRNAs designed with IRESs, we adapted a dual-luciferase reporter assay^29^, in which Firefly luciferase (Fluc) contains a weak Kozak sequence for optimal response to translation initiation alteration^33^ (Fig. 1b, Supplementary Fig. 1b), and utilized a previously characterized guide sequence to target the 3′ UTR of the Fluc mRNA^29,30^. To break away from the strict limitation for 5′ UTR-binding sites of the existing technologies, we engineered taRNAs to instead target the 3′ UTR, as mammalian 3′ UTRs are generally longer than 5’ UTRs^34^, providing more flexible choices for gRNA design and optimization, thus allowing synergetic effects and potential transcript-isoform specificity.

### IRES elements can serve as effector domains for taRNAs

Representative IRESs from each of the four major classes of viral IRESs^23^ and eukaryotic IRESs^24^ (Supplementary Fig. 1a) were selected to be evaluated as the effector domains. In our first designs, the 40-nt Fluc guide RNA (g3′1) or non-targeting gRNA (NT) and a 5-nt linker were fused to the 5′end of each IRES element, which is often flexible and tolerant of modification^35^. We expressed each putative taRNA design along with the dual-luciferase reporter in HEK293T cells and found that taRNAs with IRESs derived from viral IRES Classes 1, 2, and 3, and from endogenous mRNAs, increase targeted Fluc protein, as compared with the empty vector and non-targeting (NT) controls (Fig. 1c, Supplementary Fig. 1c), without altering relative Fluc mRNA levels (Fig. 1d, Supplementary Fig. 1d). Notably, a Class 4 IRES from Cricket Paralysis Virus (CrPV), which binds to the 40S ribosome without eIFs^36^, did not elevate Fluc expression. IRESs from hepatitis C virus (HCV)^37^, porcine teschovirus 1 (PTV-1)^38^, and encephalomyocarditis (EMCV)^39^ were then tested and verified to be effective taRNAs in HepG2 and MDA-MB-231 cells (Supplementary Fig. 1e, f), confirming the applicability of taRNAs across different cell lines. Together, these data support the hypothesis that IRESs can be engineered to recruit eIFs in *trans* at the 3′ UTR of a target mRNA, validate the taRNA design strategy for translation upregulation, and provide a set of RNA elements to serve as diverse taRNA scaffolds.

### Characterization of the guide RNA domain of taRNAs

Based on our initial screening, the taRNA built with PTV-1 IRES (PTV) was the most effective and was therefore selected for further characterization (Supplementary Fig. 1g). To probe whether the 3′UTR was the preferred gRNA landing region for taRNA, gRNAs targeting the 5′ UTR (g5′), CDS (gCDS-1 to gCDS-5), or 3′ UTR (g3′-1 and g3′-2) of Fluc mRNA were tested. Among these, the g5′, gCDS-1 and g3′-1 were previously verified to be effective binding sites for protein-based technologies^29^. Both 3′ UTR-targeted taRNAs showed the most potent activation (Fig. 1e, Supplementary Fig. 1h), thus confirming the 3′ UTR as the preferred region for taRNA targeting. We next assessed gRNA length for taRNAs, and found guide lengths between 30 nt and 50 nt were effective (Supplementary Fig. 1i). Finally, we tested the two orientations of taRNA domains and found that placement of the IRES element on the 3′ end of the guide sequence is preferred (Supplementary Fig. 1j). Taken together, using reporter transcripts, we identified and characterized both the effector and guide RNA domains of taRNAs. We next aimed to validate the technology on endogenous mRNAs in mammalian cells.

### taRNAs promote the translation of endogenous target mRNAs

**Fig. 2:**
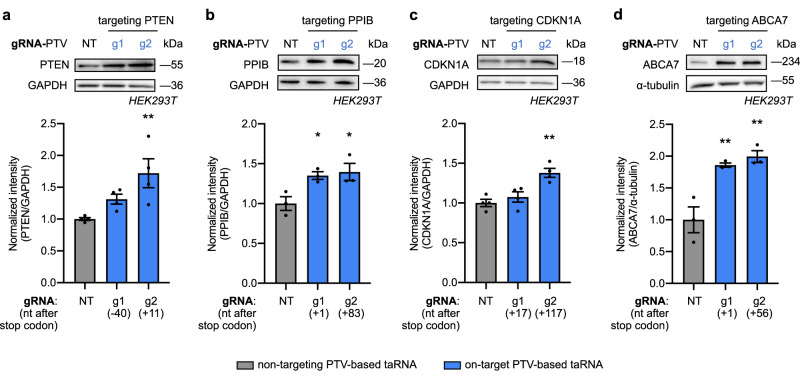
taRNAs enhance endogenous gene expression. Two gRNAs were tested in PTV-based taRNAs for each endogenous mRNA target, including **a** human PTEN; **b** human PPIB; **c** human CDKN1A; **d** human ABCA7. Each gRNA is annotated with where their last nucleotide binds relative to the stop codon on the target transcript. All experiments were done in HEK293T cells transfected with plasmids expressing the indicated taRNAs and harvested for western blots after 48 h. GAPDH or α-tubulin was used as the loading control. Representative blots were shown as top panels, and quantifications normalized to non-targeting control (NT) were shown below. All bar-graph values are shown as mean ± SEM with data points. *n* = 4 biological replicates for (**a**) and (**c**). *n* = 3 biological replicates for (**b**) and (**d**). Statistical analyses were performed using one-way ANOVA with Dunnett’s multiple comparison test vs. NT. *P < 0.05, **P < 0.01. No asterisk = not significant. The *P* value and source data are provided as a Source Data file.

A small panel of endogenous mRNAs in HEK293T cells, which contain varied Kozak sequences and are expressed and translated at a range of different levels (Supplementary Fig. 2a), was selected to validate the generality of PTV-based taRNAs. This panel of mRNAs include: the well-studied tumor suppressor, phosphatase and tensin homolog (PTEN); the highly expressed peptidylprolyl isomerase B (PPIB); the 18 kDa cell cycle regulator, Cyclin-dependent kinase inhibitor 1 (CDKN1A); and the 234 kDa transmembrane phospholipid-transporting ATPase ABCA7 (ABCA7), whose deficiency contributes to Alzheimer’s disease^40^. We designed two gRNAs on or near the 3′ UTR for each target, none of which contain stable internal secondary structures, predicted off-targets or RNA polymerase terminator sequences. The resultant taRNAs were then assayed for activation of target protein production by measuring protein levels via western blot. Across all targets, at least one of the two gRNA designs enhanced target protein production as compared to the NT control (Fig. 2a–d, Supplementary Fig. 2b–e), without affecting mRNA levels (Supplementary Fig. 2f–i). We further confirmed g2(PTEN)-PTV taRNA is also functional in a second cell line, the breast cancer MDA-MB-231 cell line (Supplementary Fig. 2j). Overall, these data demonstrate taRNAs can increase the expression of a variety of endogenous proteins, regardless of size, endogenous expression level, or localization.

To directly confirm that taRNA upregulates translation, we performed polysome profiling on HEK293T cells treated with either g2(PTEN)-PTV taRNA or empty vector (Supplementary Fig. 2k). Quantification of PTEN mRNAs in different pooled fractions—monosomes (*F3–4*), light polysomes (*F5–7*) and heavy polysomes (*F8–11*) – by RT-qPCR revealed taRNA treatment increased the relative number of PTEN mRNAs within the heavy polysome fractions, confirming that taRNA treatment results in more target transcripts undergoing active translation (Supplementary Fig. 2l). Additionally, since the total amount of PTEN mRNAs was not affected by g2(PTEN)-PTV (Supplementary Fig. 2f), the overall increase in the proportion of PTEN mRNAs that are associated with heavy polysomes may suggest that taRNA treatment promotes the engagement of translation machinery with target transcripts.

### Mechanistic interrogation via effector domain truncations

**Fig. 3:**
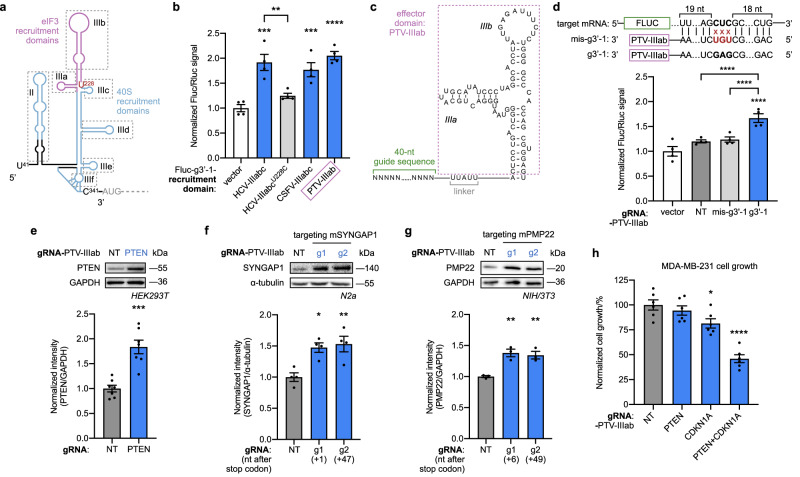
taRNAs constituted of truncated effector domains increase target protein level and manipulate cellular function. **a** Schematic of HCV IRES domains. Superscript numbers on nucleotides indicate their position on the HCV genome. U228 (red) is critical for eIF3 binding. **b** Evaluation of effector domains via dual-luciferase assay. The conserved apical domains from HCV, CSFV, and PTV-1 IRES (blue) were attached to Fluc-targeting gRNA (g3′-1). HCV-IIIabc^U228C^ (light gray) has an inactivating U228C mutation in the HCV-IRES IIIabc domain. Data were normalized to empty vector. *n* = 4 biological replicates. **c** Schematic of PTV-1 IIIab-based taRNA, with annotated domains and nucleotide sequence. **d** GAG-to-UGU mutations were introduced at the middle of Fluc-targeting gRNA in the PTV-IIIab taRNA (mis-g3′-1). This mismatched taRNA (light gray) was evaluated by dual-luciferase assay, along with original positive control (g3′-1, blue) and negative control (NT, dark gray). *n* = 4 biological replicates. **e** Representative western blot showing increased PTEN expression in HEK293T cells following transfection with g2(PTEN)-PTV-IIIab taRNA. GAPDH is the loading control. *n* = 7 biological replicates. **f** Representative western blot showing PTV-IIIab taRNA-mediated increase of SYNGAP1 protein in N2a cells. Two mSYNGAP1-targeting gRNAs (g1 and g2) were tested. α-tubulin is the loading control. *n* = 4 biological replicates. **g** Representative western blot showing PTV-IIIab taRNA-mediated increase of PMP22 protein in NIH/3T3 cells. Two mPMP22-targeting gRNAs (g1 and g2) were tested. GAPDH is the loading control. *n* = 3 biological replicates. **h** Dual upregulation of anti-proliferative proteins PTEN and p21 (gene: *CDKN1A*) by PTV-IIIab taRNAs inhibits MDA-MB-231 cell growth by 54%, measured by CCK-8 assay at 72 h after plating. *n* = 6 biological replicates. All bar-graph values are shown as mean ± SEM with data points. Statistical analyses were performed using one-way ANOVA with Sidak’s multiple comparisons test **b** vs. vector, and HCV-IIIabc vs HCV-IIIabc^U228C^; **d** vs. vector, mis-g3′-1 vs. NT, and mis-g′-1 vs. g3′-1; **f**–**h** Dunnett’s multiple comparison test vs. NT. Unpaired two-tailed Student’s *t* test was performed in (**e**). *P < 0.05, **P < 0.01, ***P < 0.001, ****P < 0.0001. The *P* value and source data are provided as a Source Data file.

Although IRESs are effective taRNA effector domains, the resulting taRNAs are still relatively large (332 nt). We therefore set out to characterize and identify the core functional components required for a truncated, IRES-derived taRNA effector domain. We started with the 302-nt HCV IRES, since it follows the same eIF-recruitment pattern as PTV-1 IRES^41^ and its domains are well-characterized^42^ (Fig. 3a). We removed everything except the IIIabc domain (HCV-IIIabc), which binds tightly to eIF3^43–45^, and found this truncated 101-nt version yields a highly active taRNA (Fig. 3b), despite missing 67% of the initial effector domain. The potency of this truncated HCV-IIIabc based taRNA were validated on endogenous PTEN in both HEK293T and MDA-MB-231 cells (Supplementary Fig 3a–c).

Based on the function of HCV-IIIabc, we postulated that eIF3 recruitment is a key mechanism utilized by HCV-based taRNAs. To test this hypothesis, a single mutation (U228C), known to disturb HCV-IIIabc structure and reduce eIF3 binding affinity to HCV IRES^43,46^, was introduced into the HCV-IIIabc taRNA (Fig. 3a). The resulting construct indeed had diminished taRNA activity, indicating eIF3 is a key endogenous target targeted by taRNAs (Fig. 3b). Since Class 3 IRESs share a conserved structural domain for eIF3 binding, including domain IIIabc for classical swine fever (CSFV) IRES^47^ and domain IIIab for PTV-1 IRES^48^, we tested whether these isolated apical domains can also serve as taRNA effectors. Both CSFV-IIIabc and PTV-IIIab were indeed effective as taRNA effector domains (Fig. 3b), providing a set of small effectors for taRNA engineering.

Aside from eIF3, we reasoned that other eIFs may also be recruited by taRNAs to activate translation. We built a second truncated taRNA based on the J–K region of EMCV IRES (EMCV-JK), which directly binds eIF4G^49^, but not eIF3^50^. This eIF4G-recruiting taRNA also enhanced both reporter and endogenous target expression (Supplementary Fig. 3d–f). Collectively, these results provide a panel of small effector domains (shorter than 100 nt) for functional taRNAs, that recruits different initiation factors (Supplementary Fig. 3g).

### Activating disease-associated genes with taRNAs

At this point, we advanced for further study with our lead candidate, the PTV-IIIab-based taRNA, which consists of a 40-nt gRNA, a 5-nt linker, and an 80 nt effector domain, resulting in a total length of 125 nt (Fig. 3c). This PTV-IIIab-based taRNA is sensitive to gRNA-binding site mismatches, as introducing three mutations (GAG to UGU) at the center of the Fluc gRNA g3′-1 (mis-g3′-1) abolished taRNA activity (Fig. 3d). Next, we confirmed the PTV-IIIab-based taRNA can activate endogenous gene expression when targeted to PTEN (Fig. 3e, Supplementary Fig. 3h).

Following validation, we applied the PTV-IIIab-based taRNA to activate protein production from haploinsufficiency-disease-relevant genes, including *SYNGAP1*, whose haploinsufficiency causes developmental delay, epilepsy, and autism^4^; and *PMP22*, whose haploinsufficiency results in Hereditary Neuropathy with Liability to Pressure Palsies (HNPP)^51^. The PTV-IIIab-based taRNA could upregulate the expression of both targets with two possible 3′ UTR-binding gRNAs relatively in mouse NIH/3T3 (Fig. 3f, Supplementary Fig. 3i) and Neuro-2a (N2a) cell lines (Fig. 3g, Supplementary Fig. 3j). Collectively, these data demonstrate taRNAs are functional across species, and have the potential to target haploinsufficiency-based disorders.

To verify that the taRNA-mediated upregulation of protein levels can produce a phenotypic effect, we used PTV-IIIab-based taRNAs to simultaneously boost the expression of both PTEN and CDKN1A in the triple-negative breast cancer cell line, MDA-MB-231 (Supplementary Fig. 3k), and monitored cellular viability. The synergistic dual upregulation of both tumor suppressor genes inhibited cancer cell growth by 54% (Fig. 3h). These data reveal that taRNAs are capable of activating more than one target in tandem, and that the upregulation from taRNA treatment drive meaningful phenotypic shifts in cells.

### Delivery of taRNAs

All experiments thus far in this study used plasmids to express taRNAs in transfected cells. However, delivery of nucleic acid-based technologies for therapy in patients require alternate strategies, most often adenovirus-associated virus (AAV) and LNPs. We therefore designed a mouse PTEN-targeting PTV-IIIab-based taRNA, which was used to compare these two delivery modalities. The mouse PTEN-targeting taRNA was packed into AAV1 and applied to NIH/3T3 cells, which upregulated PTEN expression, showing taRNAs can be delivered by AAV (Supplementary Fig. 4a).

**Fig. 4:**
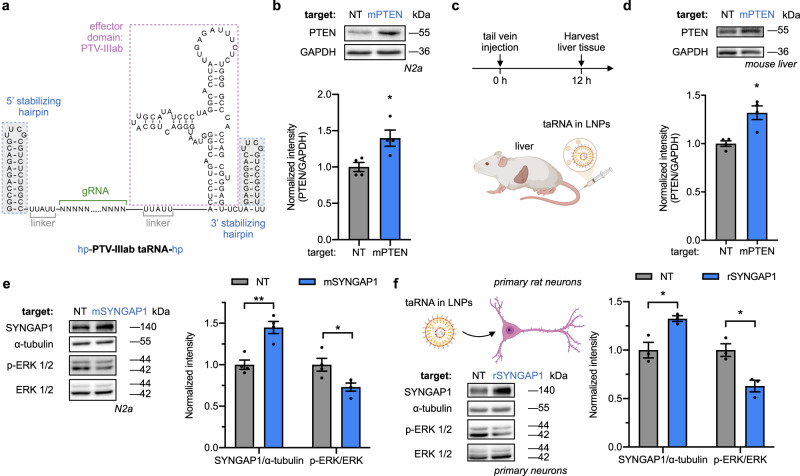
LNP delivery of taRNAs in vitro and in vivo. **a** Nucleotide sequence and secondary structure of the PTV-IIIab-based taRNA with stabilizing hairpins added at both 5′ and 3′ ends. All taRNAs in this figure utilized this stabilized form with PTV-IIIab as their effector domain, and were in vitro transcribed and delivered by LNPs. **b** Non-targeting (NT) or mouse PTEN (mPTEN)-targeting taRNA (500 ng) was delivered to N2a cells. After 12 h, the cells were lysed and PTEN protein levels measured via western blot. *n* = 4 biological replicates. **c**, **d** LNPs containing PTEN-targeting taRNAs or non-targeting taRNAs were injected into the tail vein of mice (**c**) and liver tissues were collected 12 h later. Hepatic PTEN protein levels were quantified via western blot (**d**). *n* = 4 biological replicates. LNP-packaged taRNAs targeting SYNGAP1 or non-targeting control (NT) were delivered to N2a cells (**e**) or rat primary neurons (**f**). Levels of SYNGAP1 protein and phosphorylated ERK1/2 (p-ERK1/2) were evaluated at 12 h post delivery. α-tubulin and total ERK1/2 were used for normalization as indicated. *n* = 4 biological replicates for (**e**), and *n* = 3 biological replicates for (**f**). All bar-graph values are shown as mean ± SEM with data points. Unpaired two-tailed Student’s *t* tests were performed between each group and its NT control. *P < 0.05, **P < 0.01. The *P* value and source data are provided as a Source Data file. Some elements created with BioRender.com.

While AAV delivery is important, the strength of the oligo-based taRNA design is the ability to avoid virus-based delivery systems, thus we turned our attention to delivering in vitro transcribed taRNAs packaged in clinically viable LNPs. We first confirmed that in-house generated LNPs^52^ can deliver luciferase mRNA to cell lines used in this work (Supplementary Fig. 4b). Next, to protect taRNAs from exonuclease activity, stabilizing hairpins were added to both ends of the taRNA scaffold^53^ (Fig. 4a, Supplementary Fig. 4c). This stabilized mPTEN-targeting taRNA was transcribed in vitro, encapsulated in LNPs (Supplementary Fig. 4d), and then delivered to N2a cells. Dose prescreening (Supplementary Fig. 4e) showed the optimal amount of mPTEN-targeting taRNA (500 ng) increased PTEN protein levels by 12 h post delivery (Fig. 4b, Supplementary Fig. 4f). This activation was almost gone by 24 h (Supplementary Fig. 4g), indicating, as expected, that non-modified taRNAs delivered by LNPs activate target protein production in a transient manner. We next delivered the same LNP-packaged taRNAs to live mice by intravenous (i.v.) injection to target the liver (0.5 mg/kg; Fig. 4c). At 12 h post injection, the livers were resected to analyze protein levels, which revealed the mPTEN-targeting taRNA increased the target PTEN protein compared to non-targeting taRNA control group (Fig. 4d), thereby demonstrating taRNAs can be deployed by LNPs in vivo.

We next sought to test whether taRNAs delivered by LNPs can activate SYNGAP1 translation in primary neurons. We in vitro transcribed a mouse SYNGAP1-targeting stabilized taRNA, encapsulated it in LNPs (Supplementary Fig. 5a), and identified an optimal taRNA dose in N2a cells (Supplementary Fig. 5b). The taRNA delivered to N2a cells by LNPs increased SYNGAP1 protein level at 12 h post delivery, and reduced the steady-state phosphorylation level of ERK1/2, a known downstream signaling target deactivated by SYNGAP1^54^ (Fig. 4e, Supplementary Fig. 5c). This transient effect also started to fade at 24 h post delivery (Supplementary Fig. 5d). We confirmed that primary cortical neurons from rat were amendable to LNP delivery of RNA (Supplementary Fig. 5e), and then used the same gRNA for mouse SYNGAP1 to activate rat SYNGAP1 translation, since the binding sequence is present in both species (Supplementary Fig. 5f). At 12 h post LNP delivery, the taRNAs successfully amplified SYNGAP1 expression in rat neurons and reduced steady-state ERK1/2 phosphorylation (Fig. 4f, Supplementary Fig. 5g), demonstrating the efficacy of the taRNA in primary cells.

### Optimization of taRNAs to target SYNGAP1

**Fig. 5:**
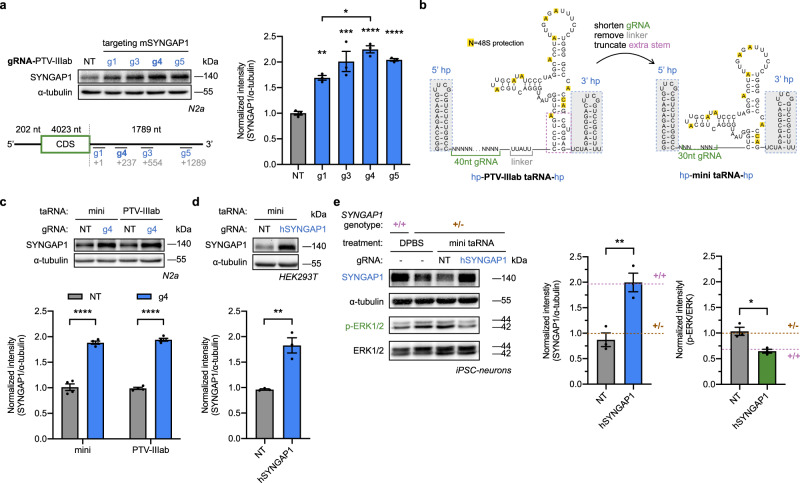
Optimized mini taRNA rescues SYNGAP1 expression in haploinsufficiency-disease cells. **a** PTV-IIIab-based taRNAs with various mouse SYNGAP1-targeting gRNAs (g1, g3, g4 and g5) were transfected into N2a cells as plasmids. The SYNGAP1 protein levels were measured by western blotting. The gRNAs are labeled with the number of nucleotides between stop codon and their landing sites (gray). The lengths of 5′ UTR, CDS and 3′ UTR of SYNGAP1 mRNA are labeled. *n* = 3 biological replicates. **b** Schematic illustrating the engineering of the PTV-IIIab-based taRNA to a minimized taRNA (mini taRNA). The detachable stabilizing hairpins on both ends (5′ hp and 3′ hp) are shadowed in gray. Nucleotides with yellow background were known to be protected upon 48S complex binding^48^. **c** mini- or PTV-IIIab-based taRNAs with NT or g4 were transfected into N2a cells as plasmids, and mouse SYNGAP1 upregulation levels were compared. g4 is the optimized guide RNA from (**a**). *n* = 4 biological replicates. **d** mini taRNA with NT or human SYNGAP1-targeting gRNA (hSYNGAP1) was transfected to HEK293T cells. *n* = 4 biological replicates. **e** Expression and functional rescue of SYNGAP1 in iPSC-derived *SYNGAP1*^+/−^ neurons. The hSYNGAP1-targeting mini taRNA and NT control were in vitro transcribed and delivered by LNPs to iPSC-neurons. At 12-h post delivery, the levels of SYNGAP1 and phosphorylated ERK1/2 were assessed by western blots. Matched iPSC-neurons from heterozygous mutant (+/−, purple) or homozygous normal (+/+, brown) individuals treated with DPBS were used as reference level (dashed lines). *n* = 3 biological replicates. For western blots, α-tubulin and total ERK1/2 were used as loading controls. Representative blots were shown, and quantifications were normalized to non-targeting control (NT). All bar-graphs are shown as mean ± SEM with data points. Statistical analyses were performed using (**a**) one-way ANOVA with Sidak’s multiple comparisons test vs. NT, and g1 vs g4; **c** two-way ANOVA with Sidak’s multiple comparisons test between g4 vs. NT in each group. Unpaired two-tailed Student’s *t* tests were performed in (**d**, **e**). *P < 0.05, **P < 0.01, ***P < 0.001, ****P < 0.0001. The *P* value and source data are provided as a Source Data file.

As a final demonstration, we aimed to optimize the taRNA platform, focused on SYNGAP1 as a testbed, for preclinical development. We started by testing whether better gRNA landing sites existed in its long 3′ UTR (1789 nt). Based on accessibility predictions (See “Methods”), three additional PTV-IIIab-based taRNAs were designed to target different sites on the 3′ UTR of mouse SYNGAP1, and tested in N2a cells. This additional screening revealed guide 4 (g4) improved PTV-IIIab-based taRNA performance, compared to the original guide 1 (g1) (Fig. 5a and Supplementary Fig. 6a).

Next, we sought to minimize the taRNA scaffold to facilitate manufacture and delivery. The guide sequence was shortened to 30 nt, which was previously found to not impact taRNA activity (Supplementary Fig. 1i), and the “linker” sequence was removed entirely. Because the nucleotides at the ends of the PTV-IIIab domain do not interact with the 48S complex (including eIF3)^48^, these extra nucleotides were also removed from the PTV-IIIab domain. Combined, these changes generated a “mini taRNA” scaffold that is only 94nt long, excluding the additional hairpins at both ends (Fig. 5b). The mini taRNA maintained efficacy for mouse SYNGAP1, comparable to the optimal mSYNGAP1-PTV-IIIab taRNA (Fig. 5c, Supplementary Fig. 6b).

To confirm the mini taRNA systems works on another target, we screened for additional gRNAs on mouse PTEN using the mini taRNA scaffold, and identified gRNAs improving taRNA efficacy on mouse PTEN activation in N2a cells (Supplementary Fig. 6c), one of which we rested in vivo (PTEN-g4), which enhanced activation for PTEN in mouse liver delivered via LNPs (Supplementary Fig. 6d). This additional example further validates the mini taRNA scaffold and demonstrates the efficacy of the system can be improved with guide RNA optimization. To confirm the mini taRNA still acts via translation regulation, a guide RNA (SYNGAP1-g4) without any effector domain was transfected into N2a cells, which was not able to increase SYNGAP1 protein level compared to negative control (non-targeting mini taRNA), while the normal mini taRNA increased SYNGAP1 expression (Supplementary Fig. 6e). This data conforms the mini taRNA acts primarily though translational regulation and not by competing with other 3’ UTR regulatory molecules.

Finally, to evaluate mini taRNA efficacy in a disease-relevant cell model, we generated iPSC-derived cortical excitatory neurons from an individual carrying a heterozygous premature stop mutation on *SYNGAP1* (c.3190C>T, p.Q1064X), and validated these *SYNGAP1*^+/−^ neurons express less SYNGAP1 protein compared to wild-type iPSC-neurons (Supplementary Fig. 6f). A mini taRNA for human SYNGAP1 upregulation was designed and shown to be effective in HEK293T cells via plasmid expression (Fig. 5d, Supplementary Fig. 6g). This mini taRNA was then in vitro transcribed with stabilized hairpins and delivered to *SYNGAP1*^+/−^ iPSC-neurons, which successfully elevated SYNGAP1 protein levels to amounts similar to iPSC-neurons derived from a healthy donor (Fig. 5e, Supplementary Fig. 6h). As a downstream signaling readout, taRNA treatment also reduced the steady-state phosphorylation of ERK1/2 (Fig. 5e), which was abnormally elevated in *SYNGAP1* haploinsufficient iPSC-neurons (Supplementary Fig. 6e). Taken together, the mini taRNA system effectively activates gene expression from a variety of targets, including haploinsufficiency-disease-relevant target in patient-derived neurons.

## Discussion

The taRNA technology is a versatile RNA-based technology to upregulate protein production of targeted endogenous mRNAs. We show that effector domains can be built from a range of IRES subdomains, suggesting that further mining of other viral and mammalian IRESs will further expand the suite of possible taRNA building blocks. The optimal guiding sequences on a target can be flexibly chosen from the 3′ UTR, and potentially from 5′ UTR (Supplementary Fig. 1h), allowing taRNAs to target, in principle, any mRNA-of-interest. This is especially important for targets whose 5′ UTR and translation initiation sites offer no suitable binding sites due to their short length or complex structure^55^, and targets whose 5′ UTR complementary sequences also bind to critical off-targets.

The taRNA technology has potential for therapeutic applications. The relatively modest level of upregulation achieved by taRNA is therapeutically beneficial for genetic haploinsufficiency diseases, since many of those disease-associated genes are dosage-sensitive and are likewise pathogenic if elevated too much^5,56^. As an example, SYNGAP1 haploinsufficiency is one of the most common causes of intellectual disability with epilepsy^57^, with no treatments available. The SYNGAP1 gene is too long for AAV-based gene replacement therapy^58^, and is not suitable for mRNA therapy, which results in overexpression. Moreover, data indicate that endogenous SYNGAP1 transcript levels vary across different types of neurons and brain regions^59,60^, thus requiring tunable rescue, depending on cell type. Ongoing efforts have focused on splice-switching oligonucleotides to avoid nonsense-mediated decay (NMD)^11,61,62^. Although effective in vitro, when tested in vivo, ASOs generate only a marginal increase in SYNGAP1 mRNA level, with unreported effects on protein levels, possibly due to the efficient clearance of SYNGAP1 NMD transcripts in neurons^62^.

The taRNA technology showcased in this study boosts protein production from productive transcripts and is therefore suitable for SYNGAP1 upregulation. Encouragingly, we found that the level of taRNA-mediated activation in *SYNGAP1* haploinsufficient neurons reached the approximate expression level of wild-type iPSC-neurons, indicating the level of activation, at least in this cell model, is in the range of therapeutic need for such a disease. The taRNA platform also offers synergistic gene-activation possibilities, if combined with technologies that increase mRNA amounts, to achieve higher protein levels as needed. Finally, because they act directly on existing transcripts, the activity of taRNAs are inherently limited to cells where SYNGAP1 mRNAs are present. Indeed, this is a key advantage of targeting translation as a gene-activation strategy.

More broadly, genome-wide association studies (GWAS) have revealed hundreds of novel disease-related genes. The taRNA platform, as a generalizable technology to upregulate specific genes, could accelerate both mechanistic studies and test the therapeutic potential of gene targets. For example, ABCA7 haploinsufficiency is associated with an increased risk for both early- and late-onset Alzheimer’s disease (AD)^63^. However, the efficient elevation of ABCA7 expression, especially in vivo, is difficult, in part because its large size (6.4 kb) exceeds the packaging capacity of AAV^58^. Activation of ABCA7 expression levels using the taRNAs described here offers a possible strategy to both probe the therapeutic potential of ABCA7 activation in animal models of AD, as well as to provide a starting point for therapeutic development.

taRNAs can immediately plug into existing AAV delivery pipelines for therapeutic development in cases where long-term gene expression changes are the goal. We also demonstrated the efficacy of LNP-delivered taRNAs as non-modified RNAs in primary neurons, iPSC-neurons, and in mouse models, proving that the utility of taRNAs can be dramatically expanded with non-viral, oligo-based delivery approaches, which are now clinically validated in the context of RNAi and other oligo technologies^64^. Given the recent successes of RNA-based therapeutics, we anticipate continuing development around oligo delivery technologies. The taRNAs fit into oligo-based systems for gene knockdown (e.g, RNAi^64^), editing (e.g. LEAPER/RESTORE^65,66^), and splicing^67^, adding an approach for targeted gene activation to the repertoire of NBT strategies. While taRNAs boosted protein expression at 12 h after delivery, the effect faded at 24 h. We built an even shorter taRNA scaffold of less than 100 nt to facilitate synthesis and chemical modifications, which in principle can enhance the stability of the system and reduce immunogenicity. These improvements in engineering, chemistry, and delivery of the taRNAs will pave a path forward toward broader clinical deployment.

## Methods

### Ethical statement

This research complies with all relevant ethical regulations. Animal studies were approved by the Institutional Animal Care and Use Committee (IACUC) at the University of Chicago (Protocol Numbers 72613 and 72016). Ethical approval for patient-derived cells was obtained from the Institutional Review Board for Baylor College of Medicine and Affiliated Hospitals (H-30480).

### Cloning

All plasmids were cloned using Gibson Assembly and sequenced by the University of Chicago Comprehensive Cancer Center DNA Sequencing and Genotyping Facility. PCR fragments for Gibson Assembly were generated using Q5 Hot Start DNA Polymerase (NEB). All IRES sequences were synthesized as gBlocks by IDT. Key plasmids used in this study are listed in Supplementary Table 1 with links to their vector maps and are available upon request.

### Mammalian cell culture and transfection

For cell culture assays, HEK293T (ATCC, CRL-3216), HepG2 (ATCC, HB-8065), MDA-MB-231 (ATCC, CRM-HTB-26), NIH/3T3 (ATCC, CRL-1658) and Neuro-2a cells (ATCC, CCL-131) were used. HEK293T are listed in the database of commonly misidentified cell lines maintained by ICLAC (http://iclac.org/databases/crosscontaminations/). Cells were maintained in Dulbecco’s Modified Eagle Medium (DMEM, l-glutamine, high glucose, sodium pyruvate, phenol red; Corning) supplemented with 10% fetal bovine serum (FBS; Gemini Benchmark), and 1× penicillin/streptomycin (P/S; Gibco/Life Technologies) in a humidified 37 °C incubator with 5% CO_2_. For all experiments, cells had undergone fewer than 20 passages. All cell lines tested negative for mycoplasma contamination. For transfections, cells were plated in full media without penicillin/streptomycin and transfected at 70% confluency 18–24 h later. The Lipofectamine 2000 (Invitrogen) reagent was used for HEK293T cells and lipofectamine LTX (Invitrogen) with Plus reagent was used for all other cell lines, according to the manufacturer’s protocols. Specifically, for endogenous targets, 500 ng taRNA plasmids were used in each well of 24-well plate and 1000 ng for 12-well plate.

### Guide RNA (gRNA) design and predication websites used

The guide RNA is the reverse-complementary RNA sequence for the binding site on a target transcript. We suggest screening several guide sequences in the relevant cell context as the best practice to find the most effective taRNAs. The basic requirements for a suitable guide RNA include: (1) a total length from 30 nt to 50 nt; (2) that it contains no transcription termination sequence for the RNA polymerase III in use; (3) that it contains no stable secondary structure by RNAfold prediction; and (4) that there are no off-targets by BLAT prediction. The preferable guide sequences should require low energy to open existing structures in binding regions, and have low interaction free energy to the target (Δ*G*_i_), indicating good affinity with binding sites on the target transcript. These predictions are performed with RNAup.

The websites used for all predictions are:

RNAfold^68^: http://rna.tbi.univie.ac.at/cgi-bin/RNAWebSuite/RNAfold.cgi

RNAup^68^: http://rna.tbi.univie.ac.at/cgi-bin/RNAWebSuite/RNAup.cgi

BLAT^69^: http://genome.ucsc.edu/cgi-bin/hgBlat

### Dual-luciferase reporter assay

To assess changes in protein levels, cells were co-transfected with 12 ng dual-luciferase reporter plasmid, and 300 ng of the indicated taRNA expression vector. About 16 h before transfection, cells were plated on 96-well plates (Corning) and allowed to grow to 70–80% confluency overnight. The next day, cells were transfected with the indicated plasmids by lipofectamine 2000 or LTX depending on the cell type. After 48 h of transfection, luminescence readouts of Firefly and Renilla luciferase were sequentially measured on a Biotek Synergy plate reader as previously described^29^ with the following modifications. First, growth media was reduced to 80 µL for every well. Then, 40 µL of 3× firefly assay buffer (Triton Lysis Buffer (150 mM Tris, 75 mM NaCl, 3 mM MgCl_2_, 0.25% Triton X-100) containing 15 mM DTT, 0.6 mM coenzyme A, 0.45 mM ATP, and 4.2 mg/mL d-luciferin) was added to lyse the cells and to provide the first substrate for firefly luciferase. After a 10 min incubation, the Firefly read was taken and 60 µL 3× Renilla assay buffer (45 mM EDTA, 30 mM sodium pyrophosphate, 1.4 M NaCl, 0.01 mM coelenterazine h (CTZ-h), 0.06 mM PTC124) was added to stop Firefly luciferase activity and provide the substrate for Renilla luciferase. The Renilla signal was taken within 5 min. All experiments conducted in six biological replicates had the highest and lowest values omitted. Firefly luciferase luminescence read were divided by the corresponding Renilla luminescence read to generate the normalized change in protein levels of the target Firefly luciferase.

### Western blotting

The treated cells were washed with PBS and lysed in RIPA buffer (50 mM Tris, 150 mM NaCl, 1% Triton X-100, 0.5% sodium deoxycholate, 0.1% SDS, 1 mM EDTA, pH 7.4) supplemented with protease inhibitors and phosphatase inhibitors (Santa Cruz sc-45045). After 10 min incubation at room temperature, the lysates were centrifuged to remove debris. Total protein concentration was measured by BCA assay (Thermo Scientific). Ten micrograms to 35 μg total protein was boiled in protein loading buffer (50 mM Tris pH 6.8, 2% SDS, 10% glycerol, 0.05% bromophenol blue, 100 mM DTT) for 5 min at 70~90 °C and loaded onto 8–12% SDS-PAGE gel according to target protein size. The total protein amount loaded was confirmed to be within linear range of detection for each antibody to detect each target protein. After stacking at 90 V, the gel was run at 140 V until the dye front reached the bottom. The proteins were transferred onto a methanol activated PVDF membrane (pore size 0.45 µm; Immobilon-P from Millipore) using semi-dry transfer apparatus (Bio-rad) or wet transfer system (Bio-rad). Membranes were blocked with 3% BSA in TBST buffer for 1 h at room temperature, incubated with primary antibody in 3% BSA-TBST at 4 °C overnight, and then washed with TBST buffer (4 × 5 min), followed by corresponding HRP-conjugated secondary antibody incubation 1 h at room temperature. The loading control GAPDH and α-tubulin were visualized using 1:2500 HRP-conjugated anti-GAPDH or anti-α-tubulin antibody. Membranes were imaged on a Fluor Chem R (Protein Simple) imager after incubation with SuperSignal West Pico PLUS chemiluminescent substrate (Thermo Scientific). For antibody dilutions and vendor information, see Table S5. For full western membrane images, see Supplementary Fig 6.

### Quantification of signal intensity on western blots

All image analysis was performed with Fiji/ImageJ. The 16-bit image from chemiluminescence channel was first set to 8-bit, then processed to subtract background using a “rolling ball” algorithm. The radius was set at 50 pixels, which is at least the size of the largest band that is not part of the background, as measured in the images. The band intensity was then measured by ImageJ within the Regions of Interest (ROIs), which is set as the same dimensions for all bands across the same image. The mean intensity is then divided by the corresponding loading control intensity, as the quantified intensity for comparation.

### RT-qPCR

Cells were plated on 96-well plates (Corning) and transfected at 70% confluency as described above for luciferase assays. Total RNA was harvested 48 h after transfection and isolated using the RNeasy Mini Kit (QIAGEN). After isolation, RNA was reversely transcribed to cDNA using the PrimeScript RT Reagent Kit (TaKaRa). All qPCR reactions were run at 20 µL volumes with at least 3 biological replicates using FastStart Essential DNA Green Master (Roche) and amplified on a LightCycler 96 Instrument (Roche). The qPCR primers were either identified based on previous publications or verified for specificity using NCBI Primer BLAST. Expression levels were calculated using the housekeeping control gene (GAPDH) cycle threshold (*C*_t_) value and the gene of interest *C*_t_ value. The relative expression level of one gene was determined by 2^−ΔCt^, where ΔCt = *C*_t_ (gene of interest) − *C*_t_ (GAPDH). Relative expression level for targeted gene was obtained upon dividing the targeted gene expression level of cells experiments treated with the on-target taRNA by those treated by the non-targeting (NT) taRNA. All qPCR primers can be found in Table S4.

### Polysome profiling and RT-qPCR of fractions

For polysome profiling, HEK293T cells were transfected with empty plasmids or PTEN-taRNA expressing vectors and cultured in two 15 cm dishes for 48 h. Then, 200 µL of cycloheximide (10 mg/ml) was added to the media at a final concentration of 100 μg/ml and incubated in 37 °C incubator for 5 min. Cells were washed twice with ice-cold PBS containing 100 μg/ml cycloheximide, collected by scraping, and after centrifugation (200 × *g* for 5 min at 4 °C), the cell pellet was resuspended in 425 µL of a hypotonic lysis buffer (5 mM Tris–HCl [pH 7.5], 2.5 mM MgCl_2_, 1.5 mM KCl, and protease inhibitor). Next, 5 μL of cycloheximide (10 mg/ml), 1 μl of 1 M dithiothreitol [DTT], 100 units of RNase inhibitor, 25 µL of 10% Triton X-100 and 25 µL of sodium deoxycholate were added to the cell suspension and vortexed for 5 second. Lysates were centrifuged at 16,000 × *g* for 7 min at 4 °C and supernatant OD were measured at 260 nm. Five hundred microliters of supernatants were adjusted according to OD and centrifuged through a 10–50% (wt/vol) sucrose gradient at 36,000 rpm for 2 h at 4 °C in an SW41Ti Rotor (Beckman Coulter). Gradients were fractionated, and optical density at 254 nm was continuously recorded by the Brandel Gradient Fractionation System (BR-188 Density Gradient Fractionation System, MD, USA). Fractions were combined for monosome-, light polysome- and heavy polysome-bound groups. RNA from each group was isolated with Trizol reagent (Invitrogen) according to the manufacturer’s protocols. RT-PCR reactions were carried out as described above.

### Cell growth assay

MDA-MB-231 cells were plated on 24-well plate 18–24 h before transfection. The control cells were transfected with 1 μg NT-PTV-IIIab taRNA plasmids, and the experimental cells were transfected with 1 μg PTEN-targeting PTV-IIIab, or 1 μg CDKN1A-targeting PTV-IIIab, or 500 ng PTEN targeting PTV-IIIab taRNA together with 500 ng p21 targeting PTV-IIIab taRNA vectors, using Lipofectamine LTX with Plus reagent (Invitrogen) per well. After 24 h of transfection, the cells were trypsinized, counted and then plated at 5 × 10^3^ cells per well in 100 μL full media without antibiotics in a 96-well plate. The cell growth was determined at 72 h post replating by Cell Counting Kit-8 (CCK-8, abcam). Briefly, 10 μL CCK-8 reagent was added to each well in 96-well plate, and the plate was incubated for 1 h at 37 °C before its absorbance was measured at 460 nm. The background without any cell was also measured in the wells filled with the 100 μL media and subtracted from each well’s read. The cell viability was all normalized to NT control group.

### AAV production and purification

HEK293T cells were seeded in 100-mm or 150-mm plates. Twenty-four hours after seeding, cells were co-transfected with AAV transfer plasmids, helper plasmids (Addgene # 112867), and Rep/Cap plasmids (Addgene # 112862 for AAV1, # 112865 for AAV9) in a 1:1:1 molar ratio using 3 µg of acidified (0.1 N HCl) polyethylenimine (PEI) per 1 µg of DNA^70^. The total amount of DNA transfected into cells in 100-mm or 150-mm plate is 13 or 36 µg, respectively. Culture media was replaced 24 h after transfection. Starting from 48 h post transfection, cell culture media was collected every day for 3 days. AAV particles were precipitated from collected media using PEG-it Virus Precipitation Solution (System Biosciences, LV810A-1) following the manufacture’s protocol, and stored at 4 °C until use within in a week.

### AAV transduction

The titer of AAV was determined by qPCR using ITR primers (Fwd: GGAACCCCTAGTGATGGAGTT, Rev: CGGCCTCAGTGAGCGA). NIH/3T3 cells were seeded in 24-well plates at 0.1 × 10^6^ cells/well at least 24 h before transduction. On the day of transduction, cell culture media was replaced with AAV particles diluted in 150 µL fresh DMEM with 10% FBS without antibiotics, at multiplicity of infection (MOI) of 500,000 vg/cell. Six hours after incubation, 500 µL fresh DMEM with 10% FBS without antibiotics was added into each well. Cells were harvested 72 h post transduction and subjected to western blot analysis.

### In-vitro transcription of mRNA and taRNA

DNA templates containing the T7 RNAP promoter were either from suitable plasmids or synthesized by IDT and were amplified by PCR prior to transcription. The template for mRNA transcription has a poly(A) tail (120 adenine nucleotides) added at its 3′ end using a long reverse primer during PCR. For a 250 µL reaction, 6 μg purified template was incubated with 1× transcription buffer (40 mM Tris-HCl, 2 mM spermidine, 10 mM NaCl), 25 mM MgCl_2_, 10 mM DTT, 40U SUPERase•In, 4 mM of each NTP, and 40 mg/mL T7 RNAP at 37 °C overnight. The next day, the resulting mixture was DNaseI digested in 1× DNaseI buffer (ThermoFisher) for 30 min at 37 °C, and then RNA was purified using RNA Clean & Concentrator-25 (RCC-25) Kits (Zymo). For mRNAs, the eluted product was 5′-capped with ScriptCap Cap 1 Capping System (Cell Script). For taRNAs, per 10 μg eluted product was treated with 25 units alkaline phosphatase, Calf Intestinal (Quick CIP, NEB) at 37 °C for 3 h, to remove its 5’-triphosphate for less toxicity and immunogenicity in cells^71^. The product was purified again with RCC-25 kit and checked for purity on 8 M urea-PAGE gel before storage or use.

### LNP formulation and characterization

Nanoparticles for mPTEN-targeting taRNAs, including for delivery to mouse liver in vivo, were produced using the GenVoy-ILM kit (Precision NanoSystems) on a NanoAssemblr Ignite device according to the manufacturer’s protocol. Briefly, 1.5 mL of taRNA at 174 μg/mL in PNI buffer provided with GenVoy-ILM kit was mixed with 0.5 mL GenVoy-ILM proprietary lipid mix under controlled conditions on the Ignite device to form RNA-LNPs, which was then diluted in 78 mL DPBS buffer and re-concentrated using Amicon Ultra-15 Centrifugal Filter Units (10,000 MWCO, MilliPore). The mean diameters and polydispersity index (PDI) of the LNPs after concentration were determined by dynamic light scattering (DLS, Wyatt DynaPro NanoStar) using 25 μL of particles. Each sample was analyzed for 10 runs and averaged. The LNP solutions were then filtered through sterile syringe filters (0.22 μm, Acrodisc) for use.

Nanoparticles for mSYNGAP1 (rSYNGAP1) -targeting taRNAs, including for primary rat neurons, were produced with the Neuro9 kit (Precision NanoSystems) on a NanoAssemblr Spark device (Precision NanoSystems). Briefly, 32 μL of taRNA at 930 μg/mL in FB1, 48 Formulation Buffer 2 (FB2, Precision NanoSystems) and 16 μL Nanoparticle mix was mixed under the control of NanoAssemblr Spark device with setting 4. The RNA-LNPs are immediately added to 96 μL FB2 to be ready for characterization and use. The mean diameters and PDI of the LNPs were also determined by DLS.

The encapsulation efficiencies and concentrations of LNPs were measured using Quant-it RiboGreen RNA Assay Kit (Invitrogen). The taRNA-LNPs were incubated either in TE buffer or in Triton buffer (1% Triton X-100 in TE buffer) for 10 min. Ribogreen reagent was then added to each incubated sample and fluorescence signal was recorded. The unencapsulated RNA amount (*F*_u_) was determined with TE buffer incubated LNPs, and the total RNA amount (*F*_t_) was the value from Triton buffer lysed samples. The encapsulation efficiency (%) = (*F*_t_ − *F*_u_)/*F*_*t*_ x 100.

### LNP delivery of taRNAs to N2a cells in vitro

About 16 h before delivery, N2a cells were plated on the 12-well plate (Corning) in full DMEM media without antibiotics, and reached 70~75% confluency at the delivery time. For each well, the indicated amount of taRNAs in LNPs were diluted with sterile DPBS buffer to make the final volume as 100 μL, which is then added dropwise to the cells. After indicated time of incubation, the cells were harvested for further analysis.

### LNP delivery of taRNAs to mouse liver in vivo

Female BALB/C mice (aged 5–6 weeks, Charles River Laboratories) received intravenous (tail vein) injections of LNPs containing 0.5 mg/kg stable hairpin-containing PTV-IIIab-based or mini taRNA (Fig. 4a), which was either non-targeting or mouse PTEN targeting. Mice were sacrificed 12 h post injection, and the liver was harvested and flash-frozen with liquid nitrogen for biochemical analysis. All animal experiments were performed following the protocols approved by the University of Chicago Institutional Animal Care and Use Committee.

### Primary neuronal culture and LNP delivery

Primary cultures of rat cortical neurons were prepared as described^72^ using Neurobasal Media (NBM), 4% (v/v) B27, and 0.125 mM l-glutamine (all from Thermo Fisher Scientific, Waltham, MA). Dissociated cortical neurons from E18 Sprague Dawley rat pups were plated in six-well plates coated with poly-D-lysine (Sigma, St Louis, MO). For western blots, 0.4 × 10^6^ cells were plated in each well. At day 8 of culture, 1 μg taRNAs encapsulated in LNPs were added and cells were incubated for 12 h before they were harvested for western blots. For imaging, the neurons were plated at 0.25 × 10^6^ cells per well, and at day 14 of culture, 1 μg GFP mRNA encapsulated by LNPs was added to each well, and the images were taken on an inverted epifluorescence microscope (Leica DMi8) with a 20× objective, a Hamamatsu Orca-Flash 4.0 camera, and a 300 W Xenon light source (Sutter Lambda XL).

### Generation and validation of induced pluripotent stem cells

An individual heterozygous for a premature termination codon mutation (c.3190C>T, p.Q1064X) in *SYNGAP1* was described previously^73^. Peripheral blood mononuclear cells from this individual were reprogrammed to induced pluripotent stem cells (iPSC) by transducing cells with Sendai virus encoding Kof4, Oct3/4, Sox2 and Myc (Cyto-Tune, ThermoFisher). Selected iPSC clonal lines were shown to be pluripotent with the Pluritest (ThermoFisher) and by immunostaining for pluripotency markers. Cell lines had a normal karyotype and were free from mycoplasma infection. Written informed consent was obtained from the parents of participant according to approved protocols by the Baylor College of Medicine Institutional Review Board.

### Neuronal differentiation from human iPSCs and LNP delivery to iPSC-neurons

Human iPSC-derived cortical excitatory neurons were generated by neurogenin-2 (NGN2) induction with modifications^74^. Briefly, human iPSCs in suspension were transduced by separate lentiviral vectors encoding neuogenin-2 driven by a tetracycline-inducible promoter (TetO-NGN2), reverse tetracycline-controlled transactivator (rtTA) and red fluorescent protein (RFP) then plated on matrigel-coated plates in mTeSR medium containing 10 µM Y27632 (StemCell technologies). Cells were maintained in a transitional medium from knockout serum replacement to neural induction medium with the supplements LDN-193189 (100 nM, Stemgent, Lexington, MA), SB431542 (10 µM, StemGent), and XAV939 (2 µM, Sigma-Aldrich, St. Louis, MO). NGN2 and RFP expression were induced by 2 µg/ml doxycycline one day prior to a 48 h selection in puromycin (2 µg/ml). After day 5, induced neuronal cells were plated over mouse glial cells cultured on poly-d-lysine/laminin coated 6-well tissue culture plates, and continually maintained in Neurobasal medium supplemented with N2, B27, BDNF (10 ng/ml, PeproTech, Rocky Hill, NJ) and doxycycline (2 µg/ml) for 33 to 55 days. At the time of treatment, 250 ng taRNAs encapsulated in LNPs or DPBS at the same volume were added to each 6-well plate. Cells were incubated for 12 h before they were harvested for western blots as described above.

### Statistics and reproducibility

All data are presented as mean ± SEM with individual data points. Statistical significance was assessed by unpaired two-tailed Student’s *t* test, one-way analysis of variance (ANOVA) or two-way analysis of variance (ANOVA) as described in the figure legends. *P* < 0.05 is considered statistically significant. Exact *P* values are provided in the source data file. All experiments were performed three or more times independently under identical or similar conditions, except when indicated in the figure legends.

### Reporting summary

Further information on research design is available in the Nature Portfolio Reporting Summary linked to this article.

### Supplementary information

Supplementary Information

Peer Review File

Reporting Summary

### Source data

Source Data

## Data Availability

Source data are provided with this paper.
