# Supplementary material for: RNA-based translation activators for targeted gene upregulation
Source: Nat Commun. 2023 Oct 26;14:6827. doi: 10.1038/s41467-023-42252-z (PMC10603104; doi:10.1038/s41467-023-42252-z)
Supplement: Supplementary file 1 — Supplementary Information [file 41467_2023_42252_MOESM1_ESM.pdf]

SUPPORTING INFORMATION:

RNA-based translational activators for targeted gene upregulation

Yang Cao[1], Huachun Liu[1], Shannon S. Lu[1], Krysten A. Jones[1], Anitha P. Govind[2], Okunola Jeyifous[2], Christine Q. Simmons[3], Negar Tabatabaei[4], William N. Green[2], Jimmy. L. Holder, Jr.[5,6], Soroush Tahmasebi[4], Alfred L. George, Jr.[3], Bryan C. Dickinson[1]*

[1]Department of Chemistry, The University of Chicago, Chicago, IL, USA
[2]Department of Neurobiology, The University of Chicago, Chicago, IL, USA
[3]Department of Pharmacology, Northwestern University Feinberg School of Medicine, Chicago, IL, USA
[4]Department of Pharmacology and Regenerative Medicine, University of Illinois College of Medicine, Chicago, IL, USA
[5]Department of Pediatrics, Baylor College of Medicine, Houston, TX, USA
[6]Jan and Dan Duncan Neurological Research Institute, Texas Children's Hospital, Houston, TX, USA

* To whom correspondence should be addressed. Email: dickinson@uchicago.edu

a

IRES	Class	Length	Initiation factors recruited
CrPV IRES	4	204 nt	NA
HCV IRES	3	302 nt	eIF3, eIF2
PTV-1 IRES	3	287 nt	eIF3, eIF2
EMCV IRES	2	463 nt	eIF4G, eIF4A, eIF4B, eIF3, eIF2
FMDV IRES	2	458 nt	eIF4G, eIF4A, eIF4B, eIF3, eIF2
PV IRES	1	635 nt	eIF4G, eIF4A, eIF4B, eIF3, eIF2
c-myc IRES	endogenous	352 nt	not confirmed

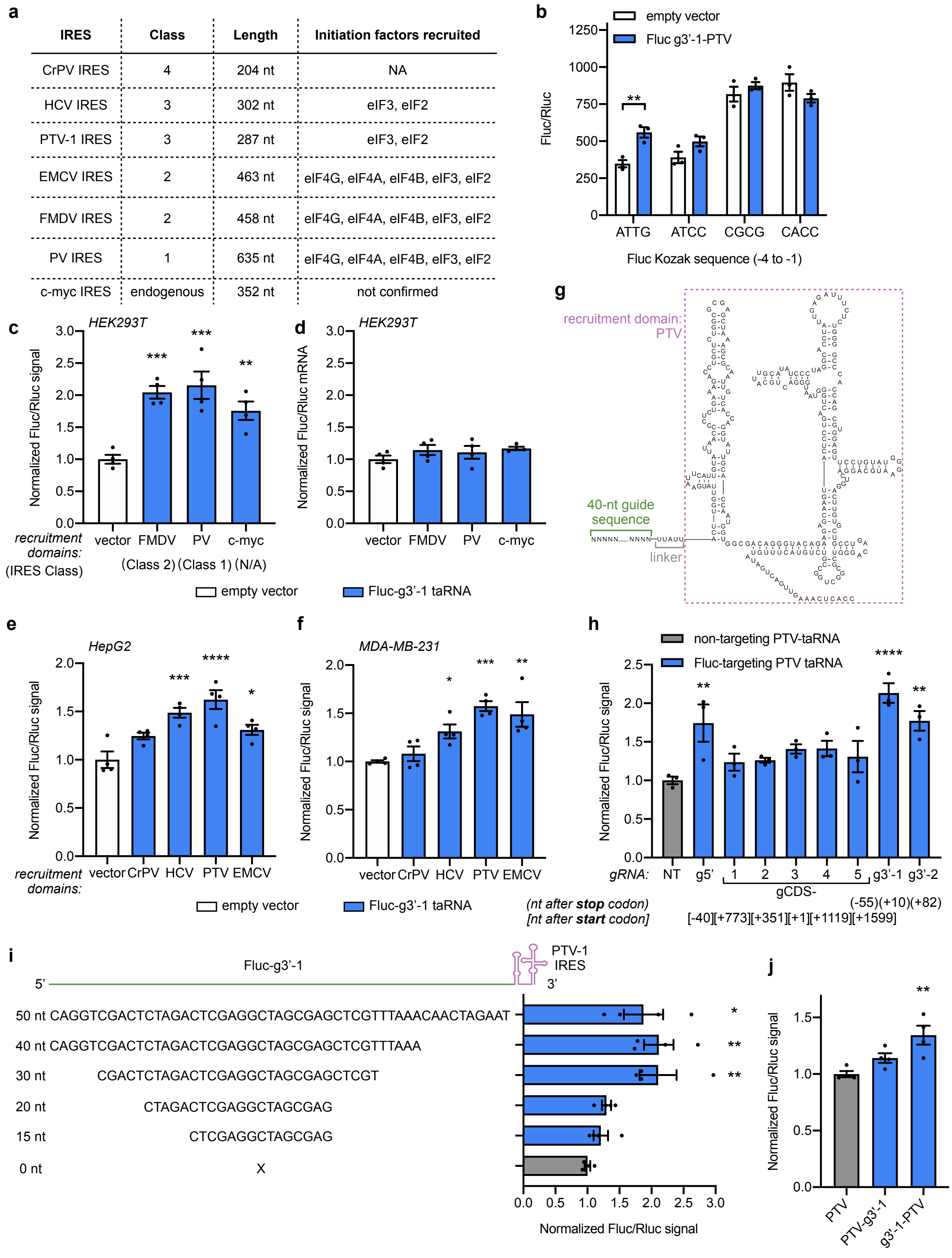

Supplementary Fig. 1 | Characterization of taRNAs using reporter. (**a**) Summary of the IRESs tested in this paper, including their classification, length and the translation initiation

factors known to be recruited by them. (**b**) Dual-luciferase reporters containing different Kozak sequences for Fluc were tested. The ATTG or ATCC containing Kozak sequence is weaker than those of CGCG and CACC, where CACC is the canonical Kozak sequence at position -4 to -1. The Fluc g3'-1- PTV taRNA (blue), together with empty vector control (white), were used to compare these reporters. n = 3 biological replicates. (**c**) Additional IRESs from FMDV, PV and human c-myc mRNA were tested as effector domains in dual-luciferase assay. Data were normalized to empty vector. n = 4 biological replicates. (**d**) RNA levels of Fluc relative to Rluc after indicated taRNA treatment were measured by RT-qPCR. Data were normalized to empty vector. n = 3 biological replicates. (**e**) The taRNAs based on CrPV, HCV, PTV-1 and EMCV IRESs were tested in HepG2 cells and (**f**) MDA-MB-231 cells. n=4 biological replicates in both (**e** and **f**). (**g**) Secondary structure of taRNA based on PTV-1 IRES shown as nucleotide sequence. (**h**) 5' UTR, CDS and 3' UTR-targeting gRNAs for Fluc were tested as PTV-based taRNA in dual-luciferase assay. Data were normalized to NT control. n = 4 biological replicates. (**i**) Evaluation of different lengths of Fluc-g3'-1 gRNA in PTV-taRNA by dual-luciferase assay. Data were normalized to 0-nt (PTV alone without gRNA, gray). (**j**) The Fluc gRNA (g3'-1) was fused at either ends of PTV-IRES and tested by dual-luciferase assay. All experiments were done in HEK293T cells in this figure unless otherwise stated. All bar-graphs are shown as mean ± SEM with data points. (**b**) Statistical analyses were performed using two-way ANOVA with Sidak's multiple comparisons test between vector and Fluc 3'-1-PTV in each Kozak sequence group. Statistical analyses were performed using one-way ANOVA with Dunnett's multiple comparison test (**c, d, e, f**) vs. vector; (**h**) vs. NT; (**i**) vs. 0 nt; (**j**) vs. PTV. No asterisk and n.s. = not significant. *P < 0.05, **P < 0.01, ***P<0.001, ****P<0.0001.

a

Target	Kozak sequence	Predicted Kozak strength (%)	mRNA abundance in HEK293 (NX*)	Protein abundance in HEK293 (ppm**)
PTEN	CCAGAC**AUG**AC	101	2.1	125
PPIB	CUGUGG**AUG**CU	26	31.1	196
ABCA7	CUCACC**AUG**GC	111	2.1	NA
CDKN1A	GGCGCC**AUG**UC	70	2.6	NA

* NX value: Normalized expression (NX) of the transcript.
** ppm: Parts per million describes abundance of each protein with reference to the entire expressed proteome.

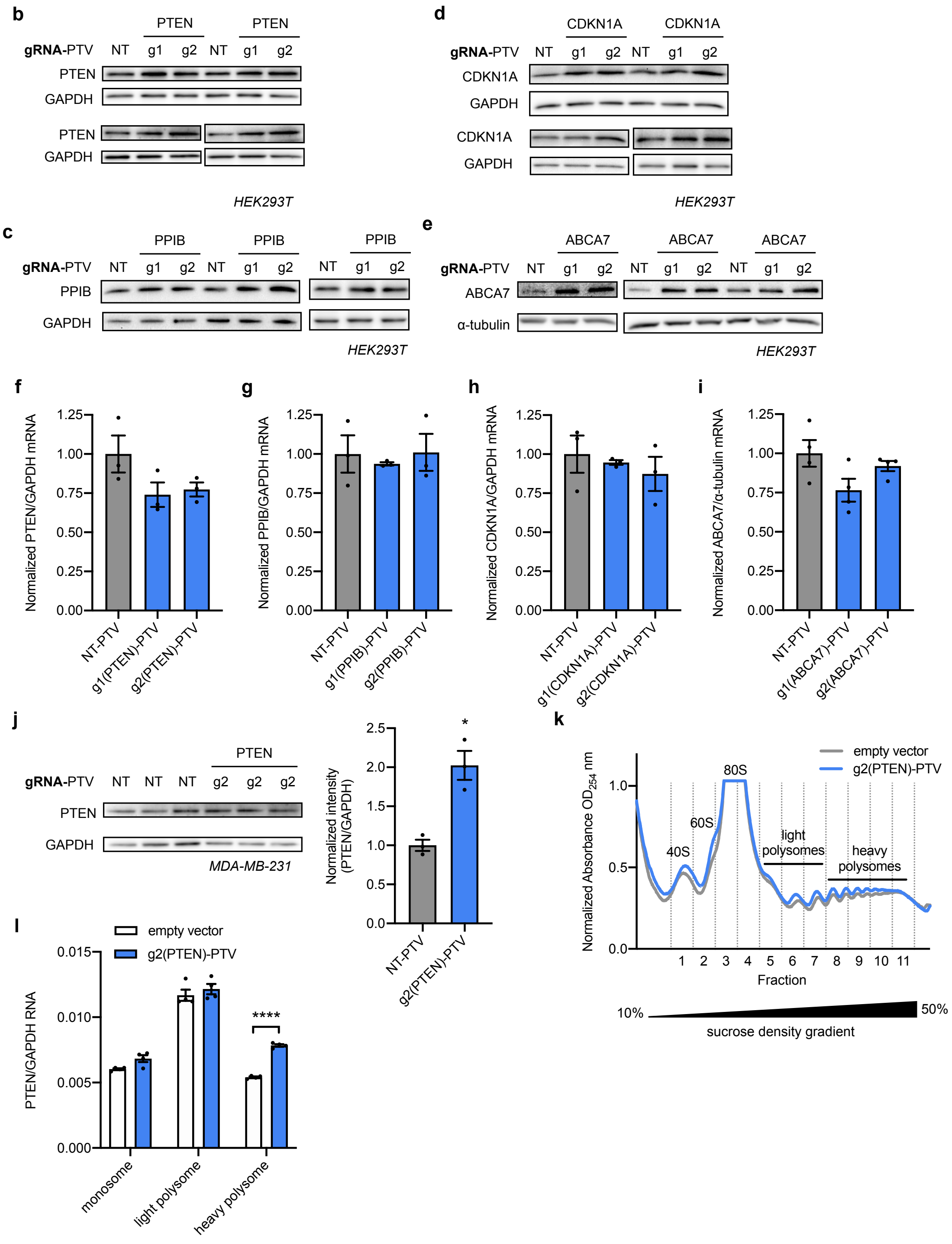

Supplementary Fig. 2 | Validation of taRNAs for increasing targeted endogenous mRNA translation. (**a**) Summary table for the characteristics of endogenous mRNAs targeted by taRNAs in **Fig. 2.** The start codons are shown in bold in Kozak sequences, and the predicted strengths are indicated as percentages when normalized to the canonical Kozak sequence[1]. The mRNA abundance data were collected from Human Protein Atlas database (proteinatlas.org), and protein abundance data were from PAXdb database[2]. Full sets of western blots used for quantification in **Fig.2** for (**b**) PTEN; (**c**) PPIB; (**d**)**,** CDKN1A; and (**e**) ABCA7 in HEK293T cells. RT-qPCR proved on-target taRNAs (blue) caused no significant changes on target RNA amount compared to non-targeting taRNAs (gray) in HEK293T cells for (**f**) PTEN; (**g**) PPIB; (**h**) CDKN1A; and (**i**) ABCA7. n=3-4 biological replicates. (**j**) Western blots and quantification for PTEN in MDA-MB-231 cells treated with either g2(PTEN)-PTV taRNA or NT control. GAPDH was the loading control. (**k**) Absorption profiles of ribosomes from HEK293T cells treated with either empty vector (gray) or g2(PTEN)-PTV (blue). Using a sucrose gradient, mRNAs were separated into 80S- (monosome), 2-4 ribosome- (light polysomes) and 5+ ribosome-bound (heavy polysomes) fractions. 40S and 60S indicate the corresponding ribosomal subunits. (**l**) Pooled fractions from (**k**) were analyzed for PTEN RNA level by RT-qPCR, normalized to GAPDH. The on-target taRNA treatment (blue) increased PTEN RNA level in overall polysome binding fractions and especially in heavy polysome fractions compared to NT control (white). n = 4 technological replicates. All bar-graph values are shown as mean ± SEM with data points. Statistical analyses were performed using one-way ANOVA (**f, g, h, i**) with Dunnett's multiple comparison test vs. NT-PTV. Unpaired two-tailed Student's *t* test was performed in (**j**). Two-way ANOVA was performed in (**l**) with Sidak's multiple comparisons test vs. empty vector. $^{*}P < 0.05$, $^{**}P < 0.01$, $^{***}P < 0.001$, $^{****}P < 0.0001$. No asterisk means not significant.

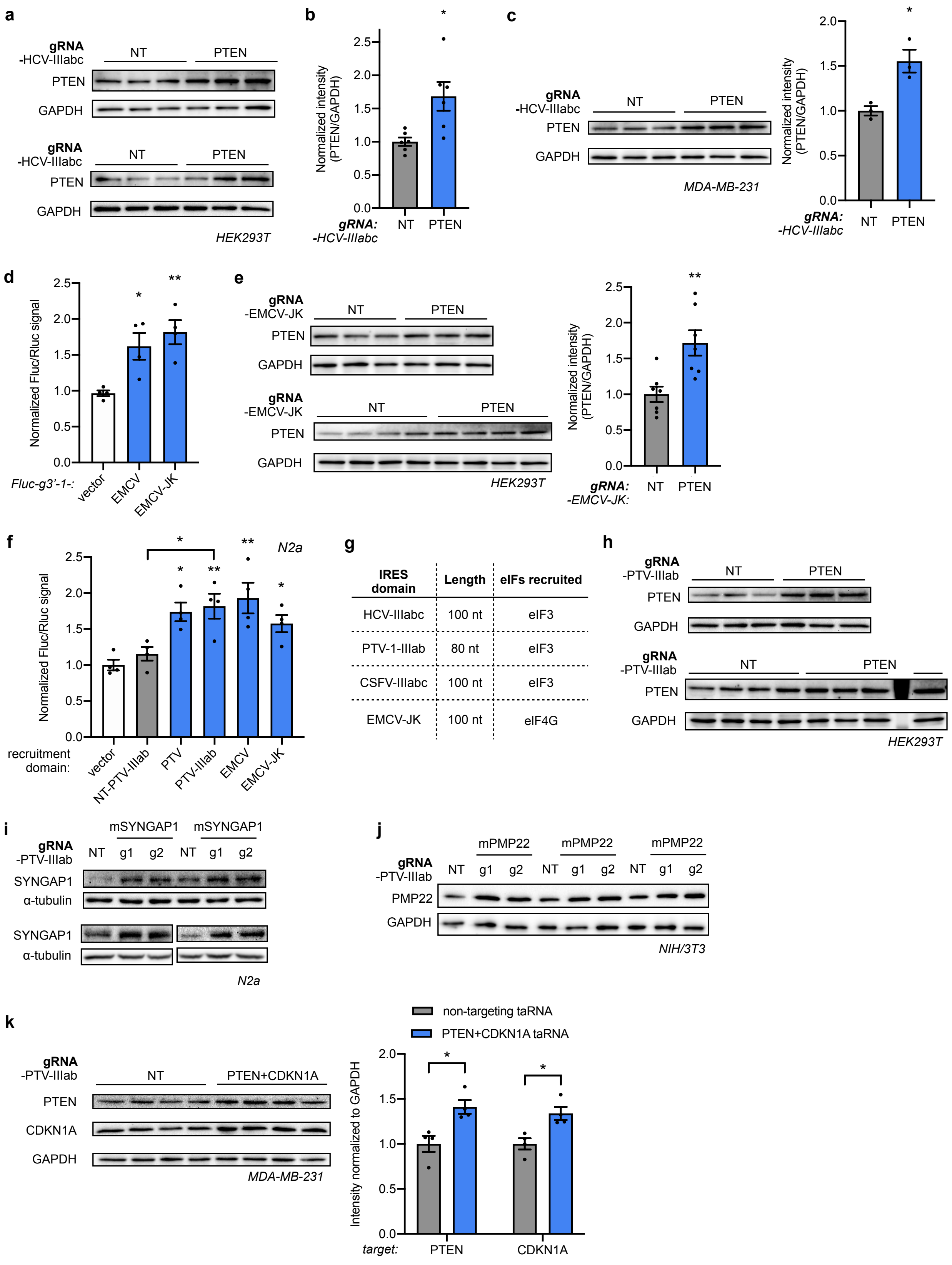

IRES domain	Length	eIFs recruited
HCV-IIIabc	100 nt	eIF3
PTV-1-IIIab	80 nt	eIF3
CSFV-IIIabc	100 nt	eIF3
EMCV-JK	100 nt	eIF4G

Supplementary Fig. 3 | Minimized IRES domains are effective taRNA recruitment domains. (**a**) Western blots and (**b**) quantification showing the increase of PTEN expression in HEK293T cells when treated with HCV-IIIabc based taRNA for PTEN compared to NT control. GAPDH was the loading control. n = 6 biological replicates. (**c**) Western blots and quantification of PTEN level with HCV-IIIabc based taRNA targeting PTEN in MDA-MB-231 cells. n = 3 biological replicates. **(d)** Full EMCV IRES and its J-K region (EMCV-JK) were tested in Fluc-targeting taRNAs. n = 4 biological replicates. (**e**) Western blots and quantification showing the increase of PTEN expression in HEK293T cells when treated with EMCV-JK based taRNA for PTEN. GAPDH was the loading control. n = 7 biological replicates. (**f**) Dual-luciferase assay confirming Fluc-targeting taRNAs from different recruitment domains are effective in mouse cell line Neuro 2A (N2a). (**g**) Table summarizing the length of minimized effector domains and the initiation factors recruited. (**h**) Full western blots used for quantification in **Fig. 3e**. (**i-j**) Full western blots used for quantification in **Fig. 3f** (**i**), and **Fig. 3g** (**j**). (**k**) Western blots and quantification of PTEN and CDKN1A increased simultaneously by PTV-IIIab based taRNAs in MDA-MB-231 cells. n = 4 biological replicates. All bar-graph values are shown as mean ± SEM with data points. Statistical analyses were performed using one-way ANOVA (**a, d**) with Dunnett's multiple comparison test vs. vector, (**f**) with Sidak's multiple comparisons test vs. empty vector, and between NT-PTV-IIIab vs. PTV-IIIab. Unpaired two-tailed Student's *t* tests were performed in (**b**, **c**, **e**, **k**). *$P < 0.05$, **$P < 0.01$, ***$P < 0.001$, ****$P < 0.0001$. No asterisk means not significant.

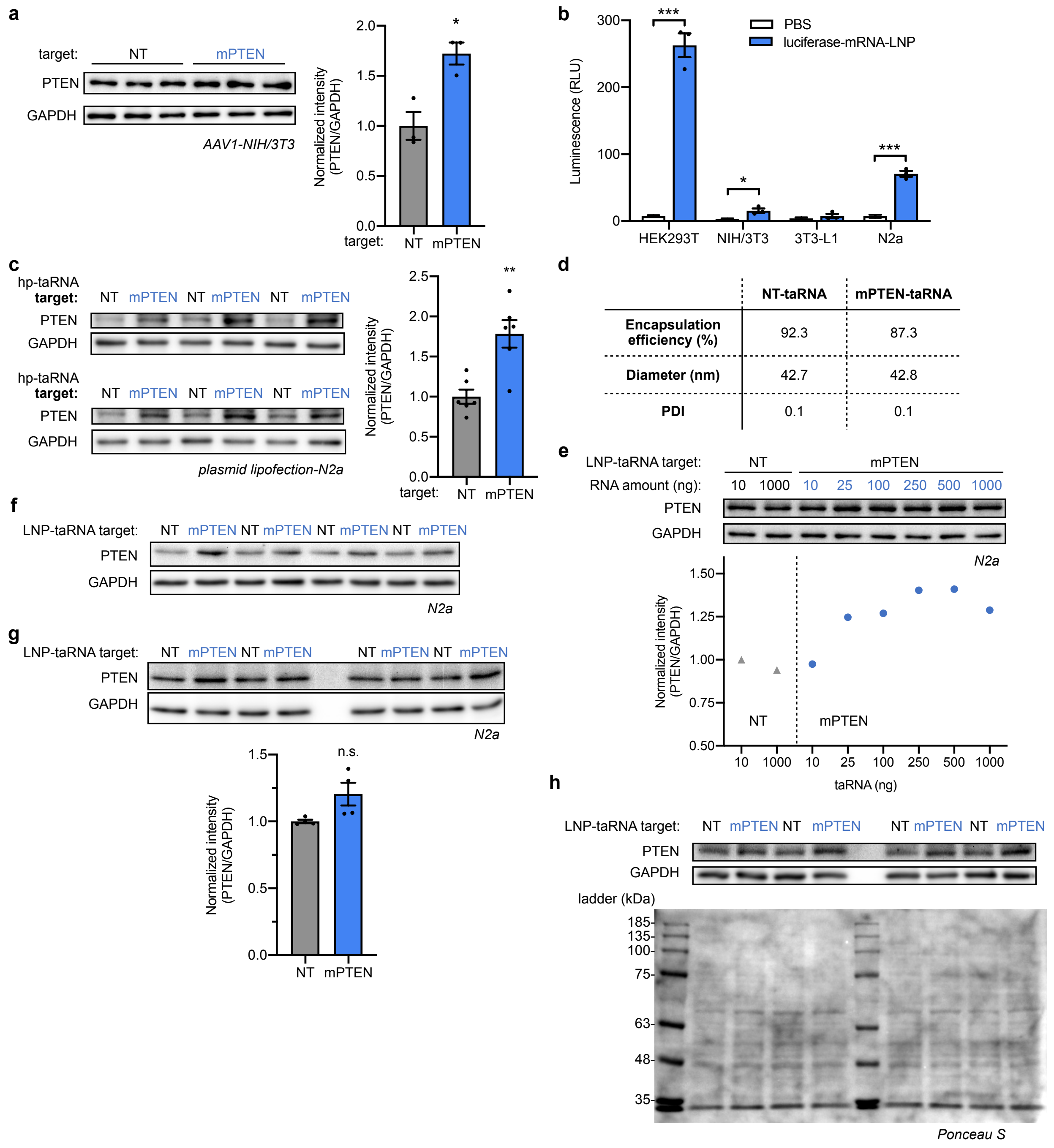

	NT-taRNA	mPTEN-taRNA
**Encapsulation efficiency (%)**	92.3	87.3
**Diameter (nm)**	42.7	42.8
**PDI**	0.1	0.1

Supplementary Fig. 4 | Delivery of mouse PTEN-targeting taRNAs. (**a**) PTV-IIIab based taRNAs targeting mouse PTEN (mPTEN) or NT were packaged into AAV1 on transfer plasmids, and transduced NIH/3T3 cells. After 48h, cells were analyzed for PTEN protein level by western blotting. (**b**) *In-vitro* transcribed Firefly luciferase mRNAs were encapsulated into LNPs, and delivered as 100ng RNA/well to different cell lines plated in 96-well plate, with DPBS as negative control. Luminescence signal was read at 24h post-delivery. (**c**) The stabilized PTV-IIIab-taRNAs with two hairpins (hp-taRNA) for mPTEN or NT were transfected into N2a cells by Lipofectamine. After 48h, the cells were lysed and PTEN level was evaluated by western blotting, with quantification shown in the right panel. (**d**) The characterization of LNPs made with NT or mPTEN-targeting taRNAs (PTV-IIIab based). The encapsulation efficiency was measured

by Ribogreen assay as described in Methods, and Dynamic light scattering (DLS) was used to measure diameter and Polydispersity Index (PDI). The stabilized PTV-IIIab-based taRNAs in LNPs were used for **Fig. S4, f, g, h.** (**e**) Two doses of NT-taRNA control (gray triangles) and 6 different doses for mPTEN-targeting taRNAs (blue circles) were delivered to N2a cells in 12-well plate for 12h before PTEN level was analyzed by western blotting. GAPDH was the loading control, and all value was normalized to that of NT-taRNA at 10 ng. (**f**) Full western blots for quantification in **Fig. 4b**. (**g**) At 24h post the delivery of NT-taRNA or mPTEN-targeting taRNA, N2a cells were lysed for PTEN protein level analysis. Western blots are shown in top panel and quantifications are shown in lower panel. (**h**) Full western blots for quantification in **Fig. 4d** are shown in top panel, and the full membrane stained with Ponceau S directly after transfer is displayed in bottom panel to show total protein loading. All bar-graph values are shown as mean ± SEM with data points. *P* values were calculated using unpaired two-tailed Student's *t* tests. **P* < 0.05, ***P* < 0.01, ****P* < 0.001, *****P* < 0.0001. No asterisk and n.s. = not significant.

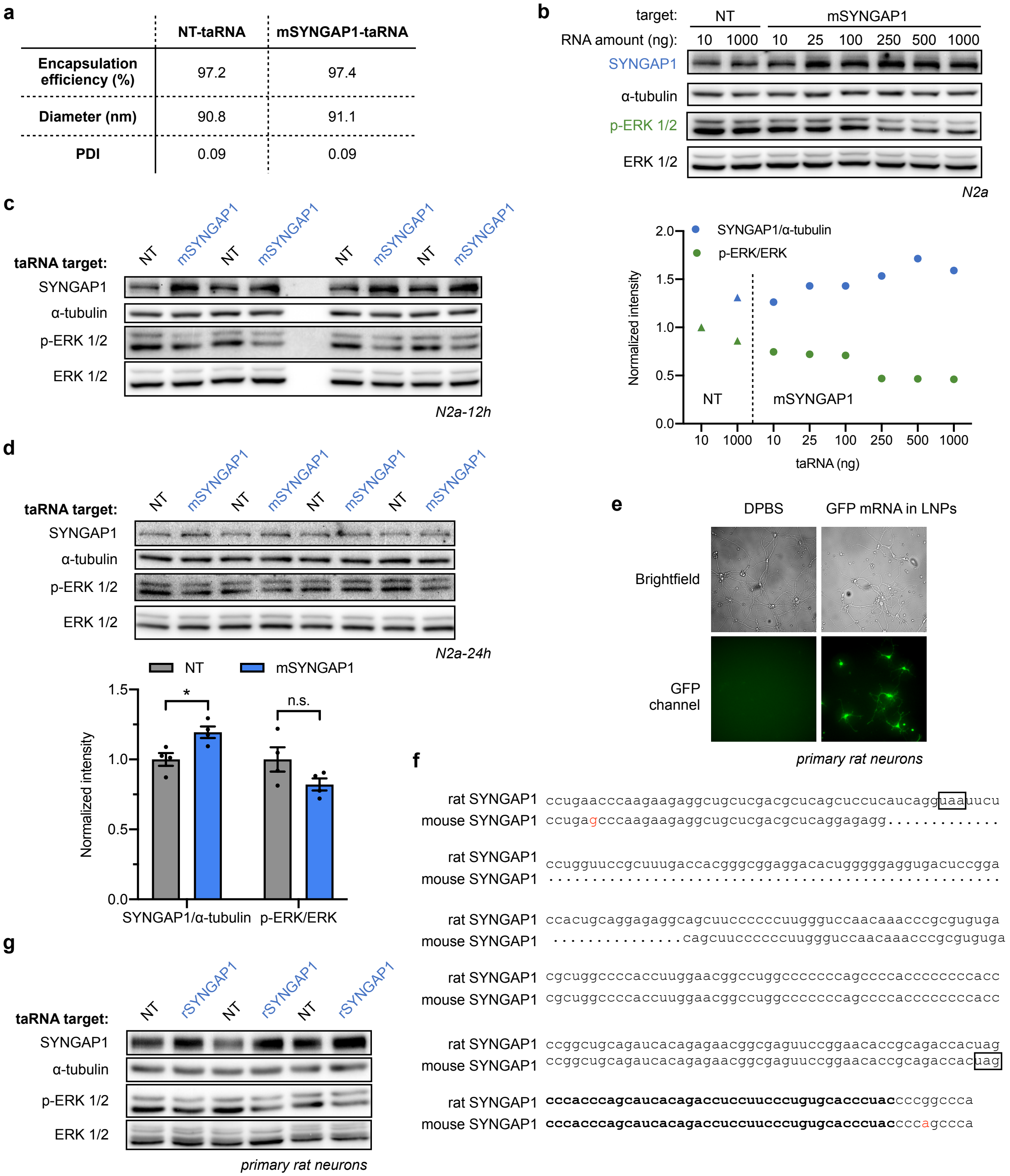

Supplementary Fig. 5 | Delivery of SYNGAP1-targeting taRNAs. The taRNAs used in this figure have stabilizing hairpins with PTV-IIIab as their effector domain, and were *in vitro*-transcribed for LNP delivery. (**a**) The characterization of LNPs made with NT or mSYNGAP1-targeting taRNAs. The encapsulation efficiency was measured by Ribogreen assay and the diameter with PDI was determined by DLS. (**b**) Two doses of NT-taRNA control (triangles) and 6 different doses for mSYNGAP1-targeting taRNAs (circles) were tested in N2a cells on 12-well plate. After 12h, SynGAP1 (blue) and phosphorylated ERK 1/2 (p-ERK 1/2, green) were quantified by western blotting, with normalization to α-tubulin and total ERK 1/2 respectively. All

values were normalized to that of NT-taRNA at 10 ng. (**c**) Full western blots used for quantification in **Fig. 4e**. (**d**) At 24h post-delivery of NT- or mSYNGAP1-targeting taRNAs, SynGAP1 and p-ERK 1/2 level were measured on western blots (top panel) in N2a cells. Quantifications are shown in lower panel. (**e**) Representative images of primary rat cortical neurons in brightfield and GFP channel, which were treated with either DPBS control or GFP mRNA encapsulated in LNPs at day 14. Images were captured and processed using the same settings between DPBS and GFP-treated groups. (**f**) Alignment of partial sequences from rat and mouse SYNGAP1 mRNA. The mismatches are colored in red and missing nucleotides are represented with dots. The stop codon of each transcript is boxed, and the nucleotides recognized by mSYNGAP1 targeting gRNA (g1) are in bold. (**g**) Full western blots used for quantification in **Fig. 4f**. The bar-graph values are shown as mean ± SEM with data points. *P* values were calculated using unpaired two-tailed student's *t* tests. $^*P < 0.05$, and n.s. means not significant.

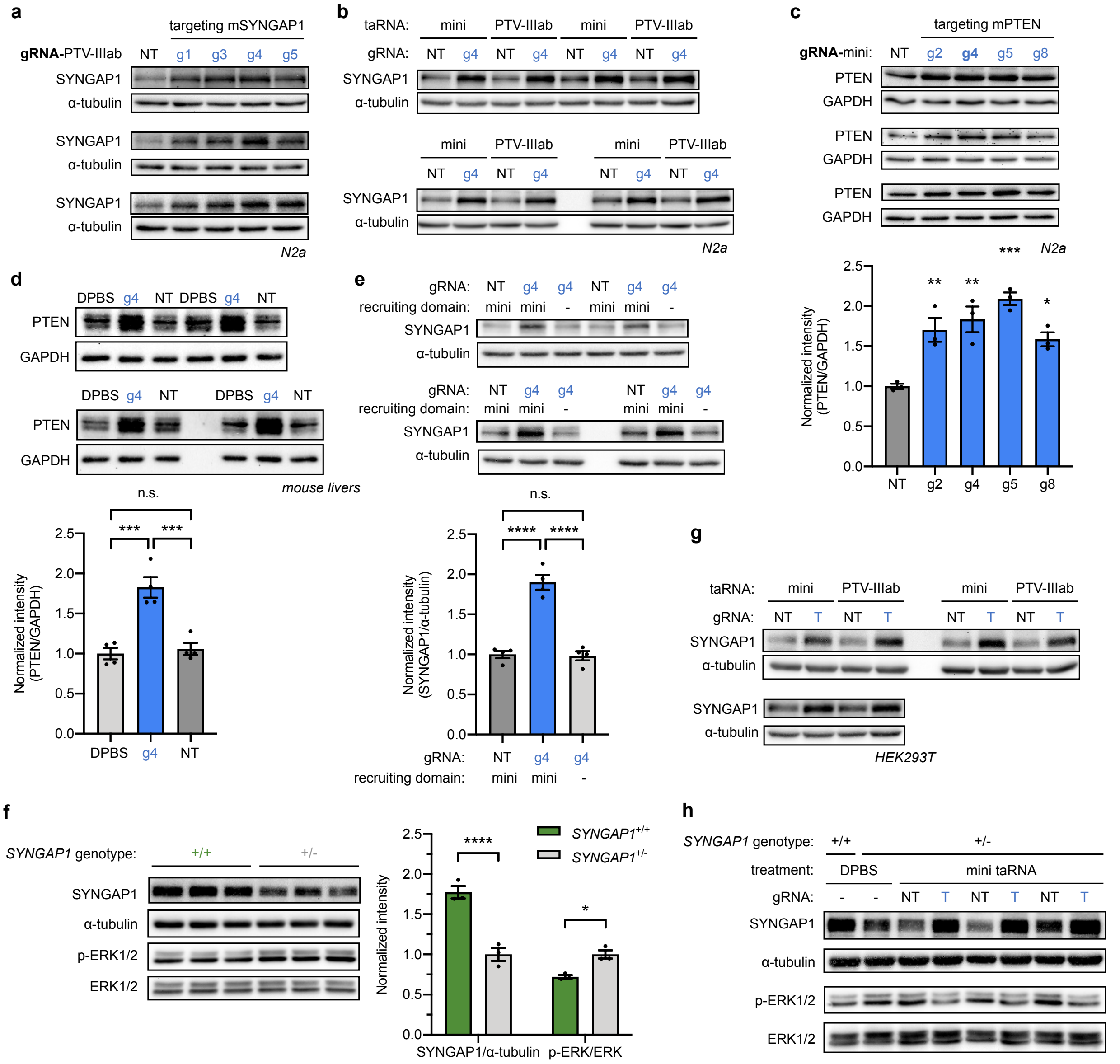

Supplementary Fig. 6 | Restoring SYNGAP1 levels in patient-derived iPSC neurons. (**a**) Full western blots used for quantification in **Fig. 5a**. (**b**) Full western blots used for quantification in **Fig. 5b**. (**c**) mini taRNAs with several guide sequences (g2, g4, g5, g8) targeting mouse PTEN (mPTEN) 3'UTR, were tested in N2a cells via plasmid transfection. PTEN protein levels were elevated by mini taRNAs. Quantification is shown below the blots, normalized to NT control. n=3 biological replicates. (**d**) mini taRNAs with non-targeting guide sequence (NT), or mouse PTEN targeting guide RNA, g4 (T), were *in-vitro* transcribed and delivered by LNPs via i.v. injection to mouse livers, as in **Fig. 4c**. At 12h post-delivery, the mouse livers were harvested and PTEN protein levels were measured by Western blotting. Quantifications are below the blots, normalized to DPBS control. N=4 biological replicates. (**e**) The SYNGAP1 guide RNA (g4) alone, without any recruitment domain ("-"), was transfected into N2a cells as plasmids. No significant change in SYNGAP1 protein level was observed compared to non-targeting mini-taRNA (NT, mini) control. g4-mini taRNA was used as a positive control. Quantification is shown below each blot, normalized to NT-mini control. n=3 biological replicates. For Western blots, GAPDH or α-tubulin was used as the loading control. (**f**)

Comparison of SYNGAP1 levels in healthy (+/+, green) or SYNGAP1 haploinsufficient (+/-, gray) iPSC- neurons, showing the *SYNGAP1*$^{+/-}$ iPSC-neurons have around ~50% intensity decrease on Western blots, parallel with SYNGAP1 protein level change, and a slight increase in steady state ERK1/2 phosphorylation level. Quantifications are on the right panel, normalized to *SYNGAP1*$^{+/+}$ level. (**g**) Full western blots used for quantification in **Fig. 5d**. (**h**) Full western blots used for quantification in **Fig. 5e**. All bar-graph values are shown as mean ± SEM with data points. Statistical analyses were performed using one-way ANOVA, (**c**) followed by Dunnett's multiple comparisons test vs. NT, (**d** and **e**) followed by Tukey's multiple comparisons. Unpaired two-tailed Student's *t* tests were performed in (**f**). *$P < 0.05$, **$P < 0.01$, ***$P < 0.001$, ****$P < 0.0001$, and n.s. means not significant.

Supplementary Table 1: Representative mammalian expression plasmids used in this study.

#	Name:	Stock No.	Description:	Plasmid Map:
1	Dual-luciferase reporter (ATTG)	47-23	PGK-ATTG-Fluc-SV40-Rluc	https://benchling.com/s/seq-ous8G6laHHS2SYCFpyxa
2	Empty vector	44-65	hU6-N/A	https://benchling.com/s/seq-VD4rAeSkTyUkjgkJCjLZ
3	Fluc g3'-1-CrPV	43-48	hU6-Fluc 3' UTR targeting gRNA-1-CrPV IRES	https://benchling.com/s/seq-kEtlcgxXm9fP3npZuCxs
4	Fluc g3'-1-HCV	42-72	hU6-Fluc 3' UTR targeting gRNA-1-HCV IRES	https://benchling.com/s/seq-z2BhpP2uZCeteXGqIiG5
5	Fluc g3'-1-PTV	39-01	hU6-Fluc 3' UTR targeting gRNA-1-PTV-1 IRES	https://benchling.com/s/seq-uBfm5RTqafo0F7mY0SQp
6	Fluc g3'-1-EMCV	42-74	hU6-Fluc 3' UTR targeting gRNA-1-EMCV IRES	https://benchling.com/s/seq-nkTvA6khZ9GPIhGlP9Tb
7	Fluc g3'-1-PV	50-50	hU6-Fluc 3' UTR targeting gRNA-1-PV IRES	https://benchling.com/s/seq-umnhNYPp9Mi74MWqBZEM
8	Fluc g3'-1-FMDV	50-51	hU6-Fluc 3' UTR targeting gRNA-1-FMDV IRES	https://benchling.com/s/seq-9xvL8e34qMYisdweZuy5
9	Fluc g3'-1-c-myc	50-52	hU6-Fluc 3' UTR targeting gRNA-1-c-myc IRES	https://benchling.com/s/seq-CeE1wlKCD5DislhLNkdL
10	PTV	44-64	hU6-PTV-1 IRES	https://benchling.com/s/seq-oMuzM3VOpdyF8hfHjWgQ
11	PTV-Fluc g3'-1	38-81	hU6-PTV-1 IRES- Fluc 3' UTR targeting gRNA-1	https://benchling.com/s/seq-xzhiX8YCAK1bLwaOwxwa
12	Fluc g3'-1-HCV-ΔII	48-28	hU6-Fluc 3' UTR targeting gRNA-1-HCV IRES without domain II	https://benchling.com/s/seq-6AjZq8bGTmjLKBPKZGiX
13	Fluc g3'-1-HCV-IIIabc	48-29	hU6-Fluc 3' UTR targeting gRNA-1-HCV IRES domain IIIabc	https://benchling.com/s/seq-qo58W1AD4phKq7Kgpuyx
14	Fluc g3'-1-HCV-IIIabc^{U228C}	50-53	hU6-Fluc 3' UTR targeting gRNA-1-HCV IRES domain IIIabc-U228C mutation	https://benchling.com/s/seq-9eHFsiOXhsN1kluNlihh
15	Fluc g3'-1-CSFV-IIIabc	50-54	hU6-Fluc 3' UTR targeting gRNA-1-CSFV IRES domain IIIabc	https://benchling.com/s/seq-SWPzsCkR0kFrLMNuWG4f

16	Fluc g3’-1-PTV-IIIab	48-37	hU6-Fluc 3’ UTR targeting gRNA-1-PTV-1 IRES domain IIIab	https://benchling.com/s/seq-ybZr3sl0ZFszka3TXzKA
17	Fluc g3’-1-EMCV-JK	51-18	hU6-Fluc 3’ UTR targeting gRNA-1-EMCV IRES JK region	https://benchling.com/s/seq-HhgIRgdPyHl5K2ahGB5F
18	AAV transfer plasmid for NT-PTV-IIIab	KJ854	pX601 AAV EF1a-EGFP-hU6-NT-PTV-IIIab	https://benchling.com/s/seq-Qq7YISO3NFm9H7h0EveC?m=slm-f4OwR9vG26s0BlrxGFM0
19	AAV transfer plasmid for mPTEN-PTV-IIIab	KJ855	pX601 AAV EF1a-EGFP-hU6-mPTEN-PTV-IIIab	https://benchling.com/s/seq-bt6EvPbkH86PwmcG2C7i?m=slm-KefpZET7lxLxddfnsXLS
20	mPTEN-PTV-IIIab with hairpins	59-21	hU6-hp-mPTEN-PTVIIIab-hp	https://benchling.com/s/seq-8Q6v9VKjl5XpEmtka1EP?m=slm-f8kyWBsE44aUlYOPmia4
21	Non-targeting mini with hairpins	62-77	hU6-hp-NT (30nt)-mini-hp	https://benchling.com/s/seq-fNT3YEjcMPqZ0usoeuph?m=slm-pi9HD0pbLazKWCfwRrT7
22	control with no recruitment domain	72-194	hU6-hp-mSYNGAP1 g4-hp	https://benchling.com/s/seq-2Tw8jxXKaCLECUkmZYvQ?m=slm-zLcpCI6drnWT1PNLqtPw

Supplementary Table 2: taRNA guide RNA sequences

#	Target:	Sequence:
1	NT (non-targeting)	UGACAGCCCACAUGGCAUUCCACUUAUCACUGGCAUCCUU
2	Fluc-g5’	GGUGGCUUUACCAACAGUACCGGAUUGCCAAGCUUGGGCU
3	Fluc-gCDS-1	UCCUCCUCGAAGCGGUACAUGAGCACGACCCGAAAGCCGC
4	Fluc-gCDS-2	CUUGCUCACGAAUACGACGGUGGGCUGGCUGAUGCCCAUG
5	Fluc-gCDS-3	AUGGCGCUGGGCCCUUCUUAAUGUUUUUGGCAUCUUCCAU
6	Fluc-gCDS-4	CUGGUUCACACCCAGUGUCUUACCGGUGUCCAAGUCCACC
7	Fluc-gCDS-5	GCCGCCCUUCUUGGCCUUAAUGAGAAUCUCGCGGAUCUUG
8	Fluc-g3’-1	CAGGUCGACUCUAGACUCGAGGCUAGCGAGCUCGUUUAAA
9	Fluc-g3’-2	GCUCAGCGGUGGCAGCAGCCAACUCAGCUUCCUUUCGGGC
10	Fluc-g3’-1-50nt	CAGGUCGACUCUAGACUCGAGGCUAGCGAGCUCGUUUAAAC AACUAGAAU
11	Fluc-g3’-1-30nt	CGACUCUAGACUCGAGGCUAGCGAGCUCGU
12	Fluc-g3’-1-20nt	CUAGACUCGAGGCUAGCGAG
13	Fluc-g3’-1-15nt	CUCGAGGCUAGCGAG
14	g1(PPIB)	CCUGCACAGACGGUCACUCAAAGAAAGAUGUCCCUGUGCC
15	g2(PPIB)	GAAUGUGAGGGGAGUGGGUCCGCUCCACCAGAUGCCAGCA
16	g1(ABCA7)	AUUCCCAGGGCCUCCCCGCGGCCCCGCAGGGGAGGGAGGC
17	g2(ABCA7)	AGCCCCUCUGCCAGCCUGAGUCCAGGGCUCCUAGGCACUC
18	g1(p21)	AGAGCGGGCCUUUGAGGCCCUCGCGCUUCCAGGACUGCAG
19	g2(p21)	GGGGGGCAGGGGGCGGCCAGGGUAUGUACAUGAGGAGGU G
20	g1(PTEN)	UCAGACUUUUGUAAUUUGUGUAUGCUGAUCUUCAUCAAAA
21	g2(PTEN)	UUAUUCAAGUUUAUUUUCAUGGUGUUUUAUCCCUCUUGAU

22	g1(mSYNGAP1)	GUAGGGUGCACAGGGAAGGAGGUCUGUGAUGCUGGGUGGG
23	g2(mSYNGAP1)	GGUGGGGUGCACAAGGAAGGAGGUCUGUGACGCUGGGUGG
24	g1(mPMP22)	CUAUGCGCGCUCAGAGCCUAGACGGACGGUGCGUCGUCGG
25	g2(mPMP22)	GUUGGUUUUGUUCUCUGGUUUCCUUCCUCCCUCCCUGUGG
26	g2(mPTEN)	CAAAACCCUGUGGAUGUAUAGGGUAAAACAAGAUUGGUCA
27	mis-Fluc-g3'	CAGGUCGACUCUAGACUCACAGCUAGCGAGCUCGUUUAAA
28	g(rSYNGAP1)	GUAGGGUGCACAGGGAAGGAGGUCUGUGAUGCUGGGUGGG
29	NT(30nt)	GCCCACAUGGCAUUCCACUUAUCACUGGCA
30	g3(mSYNGAP1)	UUCUGGGUGGGGAGAGUUAAUGUAAGAGUG
31	g4(mSYNGAP1)	AGUGAAGGGGUCUGUGUGGGGUAGGUGGUG
32	g5(mSYNGAP1)	GGGUGUAUGUAGAGGGUUAGACCGAAGGAG
33	g4(mPTEN)	UGAAGAAUUAUAAAAUAUUUAAGGAGAAAA
34	g5(mPTEN)	GCAUACUGAAUAAAUCAUUGUCAAAUUUUC
35	g8(mPTEN)	AUUUUAUCCCUCUUGAUAAGAAAAAAAAAA
36	g(hSYNGAP1)	AUUACAACAGCCAAAGAAGAGAGAAGGAAG

Supplementary Table 3: Example RNA sequences of taRNAs used in this study.

#	Name:	Sequence:
1	gRNA-HCV	NNNNNNNNNNNNNNNNNNNNNNNNNNNNNNNNNNNNNNNNuuauuUCCCCUGUGAGGAACUACUGUCUUCACGCAGAAAGCGUCUAGCCAUGGCGUUAGUAUGAGUGUCGUGCAGCCUCCAGGCCCCCCCUCCCGGGAGAGCCAUAGUGGUCUGCGGAACCGGUGAGUACACCGGAAUUGCCAGGACGACCGGGUCCUUUCUUGGAUCAAUCCCGCUCAAUGCCUGGAGAUUUGGGCGUGCCCCCGCGAGACUGCUAGCCGAGUAGUGUUGGGUCGCGAAAGGCCUUGUGGUACUGCCUGAUAGGGUGCUUGCGAGUGCCCCGGGAGGUCUCGUAGACCGUGCACC
2	gRNA-PTV	NNNNNNNNNNNNNNNNNNNNNNNNNNNNNNNNNNNNNNNNuuauuUACUUGGUUAUGAAUUCAUUGUAUUAACCCCUCUGAAAGACCUGCUCUGGCGCGAGCUAAAGCGCAAUUGUCACCAGGUAUUGCACCAAUGGUGGCGACAGGGUACAGAAGAGCAAGUACUCCUGACUGGGUAAUGGGACUGCAUUGCAUAUCCCUAGGCACCUAUUGAGAUUUCUCUGGGGCCCACCAGCGUGGAGUUCCUGUAUGGGAAUGCAGGACUGGACUUGUGCUGCCUGACAGGGUCGCGGCUGGCCGUCUGUACUUUGUAUAGUCAGUUGAAACUCACC
3	gRNA-EMCV	NNNNNNNNNNNNNNNNNNNNNNNNNNNNNNNNNNNNNNNNuuauuGAGGGCCCGGAAACCUGGCCCUGUCUUCUUGACGAGCAUUCCUAGGGGUCUUUCCCCUCUCGCCAAAGGAAUGCAAGGUCUGUUGAAUGUCGUGAAGGAAGCAGUUCCUCUGGAAGCUUCUUGAAGACAAACAACGUCUGUAGCGACCCUUUGCAGGCAGCGGAACCCCCCACCUGGCGACAGGUGCCUCUGCGGCCAAAAGCCACGUGUAUAAGAUACACCUGCAAAGGCGGCACAACCCCAGUGCCACGUUGUGAGUUGGAUAGUUGUGGAAAGAGUCAAAUGGCUCUCCUCAAGCGUAUUCAACAAGGGGCUGAAGGAUGCCCAGAAGGUACCCCAUUGUAUGGGAUCUGAUCUGGGGCCUCGGUGCACAUGCUUUACAUGUGUUUAGUCGAGGUUAAAAAAACGUCUAGGCCCCCCGAACCACGGGGACGUGGUUUUCCUUUGAAAAACACGAUGAUAA

4	gRNA-HCV-IIIabc	NNNNNNNNNNNNNNNNNNNNNNNNNNNNNNNNNNNNNNNNuuauuUCUGCGGAACCGGUGAGUACACCGGAAUUGCCAGGACGACCGGGUCCUUUCUUGGAUCAAUCCCGCUCAAUGCCUGGAGAUUUGGGCGUGCCCCCGCGAGA
5	gRNA-PTV-IIIab	NNNNNNNNNNNNNNNNNNNNNNNNNNNNNNNNNNNNNNNNuuauuACUCCUGACUGGGUAAUGGGACUGCAUUGCAUAUCCCUAGGCACCUAUUGAGAUUUCUCUGGGGCCCACCAGCGUGGAGU
6	gRNA-EMCV-JK	NNNNNNNNNNNNNNNNNNNNNNNNNNNNNNNNNNNNNNNNuuauuGGGGCUGAAGGAUGCCCAGAAGGUACCCCAUUGUAUGGGAUCUGAUCUGGGGCCUCGGUGCACAUGCUUUACAUGUGUUUAGUCGAGGUUAAAAAAACGUCUAGGCCCC
7	hairpin-gRNA-PTV-IIIab-hairpin	ggccagagacguucgcgucucuggccuuauuNNNNNNNNNNNNNNNNNNNNNNNNNNNNNNNNNNNNNNNNuuauuACUCCUGACUGGGUAAUGGGACUGCAUUGCAUAUCCCUAGGCACCUAUUGAGAUUUCUCUGGGGCCCACCAGCGUGGAGUucuagagcggacuucgguccgcuuuu
8	hairpin-gRNA-mini-IIIab-hairpin	ggccagagacguucgcgucucuggccuuauuNNNNNNNNNNNNNNNNNNNNNNNNNNNNNNCUGGGUAAUGGGACUGCAUUGCAUAUCCCUAGGCACCUAUUGAGAUUUCUCUGGGGCCCACCAGUCUAGAgcggacuucgguccgcuuuu

Supplementary Table 4: RT primers

#	Name:	Sequence:
1	Fluc-qPCR-for	GGATGCTCTCCAGTTCGGCT
2	Fluc-qPCR-rev	ACCAGCGCGGCGAGCTGT
3	Rluc-qPCR-for	CTATTGTCGAGGGAGCTAAGAAG
4	Rluc-qPCR-rev	GCTCTTGATGTACTTACCCATTTC
5	GAPDH-qPCR-for	GTCTCCTCTGACTTCAACAGCG
6	GAPDH-qPCR-rev	ACCACCCTGTTGCTGTAGCCAA
7	PPIB-qPCR-for	AACGCAGGCAAAGACACCAACG
8	PPIB-qPCR-rev	TCTGTCTTGGTGCTCTCCACCT
9	PTEN-qPCR-for	TGAGTTCCCTCAGCCGTTACCT
10	PTEN-qPCR-rev	GAGGTTTCCTCTGGTCCTGGTA
11	ABCA7-qPCR-for	CACTCTTCCGAGAGCTAGACAC
12	ABCA7-qPCR-rev	CTCCATATCTGTGTCCGCAGCA
13	CDKN1A-qPCR-for	AGGTGGACCTGGAGACTCTCAG
14	CDKN1A-qPCR-rev	TCCTCTTGGAGAAGATCAGCCG

Supplementary Table 5: Antibodies used in this study

#	Target:	Vendor:	Catalogue number	Dilution:
1	GAPDH (HRP)	Proteintech	HRP-60004	1:5000
2	α-Tubulin (HRP)	Proteintech	HRP-66031	1:5000
3	ABCA7	Proteintech	25339-1-AP	1:1000
4	PTEN	Santa Cruz Biotechnology	sc-7974	1:1000
5	PMP22	Santa Cruz Biotechnology	sc-515199	1:1000
6	PPIB	Santa Cruz Biotechnology	sc-130626	1:1000

7	CDKN1A	Santa Cruz Biotechnology	sc-6246	1:1000
8	SynGAP1	Invitrogen	PA1-046	1:1000
9	Phospho-p44/42 MAPK (Erk1/2)	Cell Signaling Technology	4370T	1:2000
10	p44/42 MAPK (Erk1/2)	Cell Signaling Technology	4695	1:2000
11	mouse IgG H&L (HRP)	Abcam	ab6728	1:5000
12	rabbit IgG H&L (HRP)	Abcam	ab6721	1:5000

Supplementary References

1. Noderer, W.L. et al. Quantitative analysis of mammalian translation initiation sites by FACS-seq. *Mol Syst Biol* **10**, 748 (2014).
2. Wang, M., Herrmann, C.J., Simonovic, M., Szklarczyk, D. & von Mering, C. Version 4.0 of PaxDb: Protein abundance data, integrated across model organisms, tissues, and cell-lines. *Proteomics* **15**, 3163-3168 (2015).
