# Supplementary material for: RNA-based translation activators for targeted gene upregulation
Source: Nat Commun. 2023 Oct 26;14:6827. doi: 10.1038/s41467-023-42252-z (PMC10603104; doi:10.1038/s41467-023-42252-z)
Supplement: Supplementary file 3 — Reporting Summary [file 41467_2023_42252_MOESM3_ESM.pdf]

nature portfolio

Corresponding author(s): Bryan Dickinson

Last updated by author(s): 2023_9_20

Reporting Summary

Nature Portfolio wishes to improve the reproducibility of the work that we publish. This form provides structure for consistency and transparency in reporting. For further information on Nature Portfolio policies, see our Editorial Policies and the Editorial Policy Checklist.

Statistics

For all statistical analyses, confirm that the following items are present in the figure legend, table legend, main text, or Methods section.

n/a	Confirmed	
☐	☒	The exact sample size (*n*) for each experimental group/condition, given as a discrete number and unit of measurement
☐	☒	A statement on whether measurements were taken from distinct samples or whether the same sample was measured repeatedly
☐	☒	The statistical test(s) used AND whether they are one- or two-sided<br>*Only common tests should be described solely by name; describe more complex techniques in the Methods section.*
☐	☒	A description of all covariates tested
☐	☒	A description of any assumptions or corrections, such as tests of normality and adjustment for multiple comparisons
☐	☒	A full description of the statistical parameters including central tendency (e.g. means) or other basic estimates (e.g. regression coefficient) AND variation (e.g. standard deviation) or associated estimates of uncertainty (e.g. confidence intervals)
☐	☒	For null hypothesis testing, the test statistic (e.g. *F*, *t*, *r*) with confidence intervals, effect sizes, degrees of freedom and *P* value noted<br>*Give P values as exact values whenever suitable.*
☒	☐	For Bayesian analysis, information on the choice of priors and Markov chain Monte Carlo settings
☒	☐	For hierarchical and complex designs, identification of the appropriate level for tests and full reporting of outcomes
☒	☐	Estimates of effect sizes (e.g. Cohen's *d*, Pearson's *r*), indicating how they were calculated

Our web collection on statistics for biologists contains articles on many of the points above.

Software and code

Policy information about availability of computer code

Data collection	No software was used.
Data analysis	ImageJ/FjJi software version 2.14.0/1.54f; GraphPad Prism software version 10.0.0 (131).

For manuscripts utilizing custom algorithms or software that are central to the research but not yet described in published literature, software must be made available to editors and reviewers. We strongly encourage code deposition in a community repository (e.g. GitHub). See the Nature Portfolio guidelines for submitting code & software for further information.

Data

Policy information about availability of data

All manuscripts must include a data availability statement. This statement should provide the following information, where applicable:

- Accession codes, unique identifiers, or web links for publicly available datasets
- A description of any restrictions on data availability
- For clinical datasets or third party data, please ensure that the statement adheres to our policy

The data generated in this study are provided in the Supplementary Information and Source Data file. Key vectors will be made available through Addgene.

Research involving human participants, their data, or biological material

Policy information about studies with human participants or human data. See also policy information about sex, gender (identity/presentation), and sexual orientation and race, ethnicity and racism.

Reporting on sex and gender	Sex or gender analysis was not needed for this study.
Reporting on race, ethnicity, or other socially relevant groupings	Socially relevant groups analysis was not needed for this study.
Population characteristics	No population characteristics analysis was needed for this study.
Recruitment	The participating subject was recruited within a dedicated Primary Synaptopathy clinic at Texas Children's Hospital in Houston, Texas.
Ethics oversight	Ethic approval for patient-derived cells was obtained from the Institutional Review Board for Baylor College of Medicine and Affiliated Hospitals (H-30480).

Note that full information on the approval of the study protocol must also be provided in the manuscript.

Field-specific reporting

Please select the one below that is the best fit for your research. If you are not sure, read the appropriate sections before making your selection.

☒ Life sciences ☐ Behavioural & social sciences ☐ Ecological, evolutionary & environmental sciences

For a reference copy of the document with all sections, see nature.com/documents/nr-reporting-summary-flat.pdf

Life sciences study design

All studies must disclose on these points even when the disclosure is negative.

Sample size	No statistical methods were used to predetermine sample sizes. Our sample size are comparable to similar studies, and statistic significant.
Data exclusions	Data were excluded from analysis only in the instance of technical failure.
Replication	All data were acquired from at least three biological replicates. Data were reproduced by different people.
Randomization	Mice were randomly selected for injection with control or treatment. In vitro experiments were not randomized.
Blinding	No blinding is needed because the samples were analyzed by western blots with internal control.

Reporting for specific materials, systems and methods

We require information from authors about some types of materials, experimental systems and methods used in many studies. Here, indicate whether each material, system or method listed is relevant to your study. If you are not sure if a list item applies to your research, read the appropriate section before selecting a response.

Materials & experimental systems

n/a	Involved in the study	
☐	☒	Antibodies
☐	☒	Eukaryotic cell lines
☒	☐	Palaeontology and archaeology
☐	☒	Animals and other organisms
☒	☐	Clinical data
☒	☐	Dual use research of concern
☒	☐	Plants

Methods

n/a	Involved in the study	
☒	☐	ChIP-seq
☒	☐	Flow cytometry
☒	☐	MRI-based neuroimaging

Antibodies

Antibodies used	Anti-GAPDH (HRP) (HRP-60004; Proteintech; 1:5000)<br>Anti-α-Tubulin (HRP) (HRP-66031; Proteintech; 1:5000)<br>Anti-ABCA7 (25339-1-AP; Proteintech; 1:1000)

Anti-PTEN (sc-7974; Santa Cruz Biotechnology; 1:1000)
Anti-PMP22 (sc-515199; Santa Cruz Biotechnology; 1:1000)
Anti-PPIB (sc-130626; Santa Cruz Biotechnology; 1:1000)
Anti-CDKN1A (sc-6246; Santa Cruz Biotechnology; 1:1000)
Anti-SynGAP1 (PA1-046; Invitrogen; 1:1000)
Anti-Phospho-p44/42 MAPK (Erk1/2) (4370T; Cell Signaling Technology; 1:2000)
Anti-p44/42 MAPK (Erk1/2) (4695; Cell Signaling Technology; 1:2000)
Anti-Mouse IgG H&L (HRP) (ab6728; Abcam; 1:5000)
Anti-Rabbit IgG H&L (HRP) (ab6721; Abcam; 1:5000)

Validation

All antibodies are are widely used and have been validated for Western blots by manufacturers with statement in their product information.

Eukaryotic cell lines

Policy information about cell lines and Sex and Gender in Research

Cell line source(s)	HEK293T (CRL-3216), HepG2 (HB-8065), MDA-MB-231 (CRM-HTB-26), NIH/3T3 (CRL-1658) and Neuro-2a cells (CCL-131) were all purchased from ATCC. Patient-derived cells
Authentication	All cell lines were authenticated by ATCC.
Mycoplasma contamination	All cell lines tested negative for mycoplasma contamination.
Commonly misidentified lines (See ICLAC register)	HEK293T is listed in the database of commonly misidentified cell lines. This cell line is used as a screening platform, whose identity is not relevant.

Animals and other research organisms

Policy information about studies involving animals; ARRIVE guidelines recommended for reporting animal research, and Sex and Gender in Research

Laboratory animals	Female BALB/C mus musculus (aged 5-6 weeks) were used in this work. E18 Sprague Dawley rats were used to isolate cortical neurons.
Wild animals	No wild animals were used.
Reporting on sex	No sex analysis was performed.
Field-collected samples	No field-collected samples were involoved.
Ethics oversight	Animal studies were approved by the Institutional Animal Care and Use Committee (IACUC) at the University of Chicago (Protocol Number: 72613, and 72016).

Note that full information on the approval of the study protocol must also be provided in the manuscript.
