# Supplementary material for: RNA-based translation activators for targeted gene upregulation
Source: Nat Commun. 2023 Oct 26;14:6827. doi: 10.1038/s41467-023-42252-z (PMC10603104; doi:10.1038/s41467-023-42252-z)
Supplement: Supplementary file 2 — Peer Review File [file 41467_2023_42252_MOESM2_ESM.pdf]

nature portfolio

Peer Review File

RNA-based translation activators for targeted gene upregulation

Open Access This file is licensed under a Creative Commons Attribution 4.0 International License, which permits use, sharing, adaptation, distribution and reproduction in any medium or format, as long as you give appropriate credit to the original author(s) and the source, provide a link to the Creative Commons license, and indicate if changes were made. In the cases where the authors are anonymous, such as is the case for the reports of anonymous peer reviewers, author attribution should be to 'Anonymous Referee' followed by a clear attribution to the source work. The images or other third party material in this file are included in the article's Creative Commons license, unless indicated otherwise in a credit line to the material. If material is not included in the article's Creative Commons license and your intended use is not permitted by statutory regulation or exceeds the permitted use, you will need to obtain permission directly from the copyright holder. To view a copy of this license, visit http://creativecommons.org/licenses/by/4.0/.

Editorial Note: This manuscript has been previously reviewed at another journal that is not operating a transparent peer review scheme. This document only contains reviewer comments and rebuttal letters for versions considered at Nature Communications.

REVIEWERS' COMMENTS

Reviewer #2 (Remarks to the Author):

I have reviewed this exact manuscript previously and do not have any additional comments. It is an interesting manuscript and I strongly feel that it merits publication.

I understand that Western blot quantification issues has been brough up by another reviewer. This approach is not necessarily a drawback, because ELISAs and dot blots may also have different background levels in different wells/slots, but as opposed to Westerns such backgrounds are not obvious and cannot be factored into result evaluation.

Furthermore, ELISAs and dot blots do not necessarily indicate which particular band/protein is being quantified, so the capture/detection antibody cross-reactivity remains hidden.

HPLC quantification has a number of potential technical problems with peak width determination, background assessment, protein integrity etc.

Also, the Westerns in this manuscripts are in fact generally of good quality and with clear differences between variants.
